# Uncovering resistance pathways to first- and last-line antibiotics in Mycobacterium tuberculosis populations

**DOI:** 10.1099/mgen.0.001723

**Published:** 2026-05-19

**Authors:** Prapaporn Srilohasin, Jasmine M. Williams, Aidan P. Tay, Brodie F. Gillieatt, Daniel R. Pascoe, Ram P. Maharjan, Angkana Chaipraset, Pravech Ajawatanawong, Amy K. Cain

**Affiliations:** 1 Department of Microbiology, Faculty of Medicine Siriraj Hospital, Mahidol University, Bangkok, Thailand; 2 School of Natural Sciences, Macquarie University, Sydney, NSW, Australia; 3 ARC Centre of Excellence in Synthetic Biology, Macquarie University, Sydney, NSW, Australia; 4 Australian Genome Foundry, Macquarie University, Sydney, NSW, Australia

**Keywords:** antibiotic resistance, directed evolution, linezolid, *Mycobacterium tuberculosis*, rifampicin, tuberculosis

## Abstract

Tuberculosis (TB), caused by *Mycobacterium tuberculosis*, is one of the world’s deadliest diseases, currently responsible for ~1.5 million deaths per year and rising. Recently, rifampicin-resistant *M. tuberculosis* was designated as a critical priority pathogen status by the World Health Organization. However, few controlled laboratory studies are available that systematically assess the molecular drivers of antibiotic resistance development in TB. In this study, we paired laboratory-directed evolution and population-level deep-sequencing approaches to map the evolutionary pathways taken by *M. tuberculosis* to develop resistance to first- and last-line therapies (rifampicin and linezolid) and then characterized *de novo* resistance mutation occurrence over time. We demonstrated that the majority of *M. tuberculosis* populations readily acquire mutations in genes commonly found in rifampicin- and linezolid-resistant clinical isolates (*rpoB* and *rplC*). However, we also identified mutations in six genes, mostly present in subpopulations (17–41%) and not previously linked to rifampicin or linezolid resistance, including four associated with rifampicin resistance (*Rv0052*, *ppsD*, *ppsE* and *mptC*) and two associated with linezolid resistance (*glpK* and *echA12*). The *ppsD*, *glpK* and *mptC* mutations were also identified in published individual sequencing reads of antibiotic-resistant clinical isolates. Further investigation of the identified resistance determinants *ppsD*/*E* established that mutations in these genes appear to mediate resistance across multiple species, with an *Escherichia coli* mutant of the ortholog (*fabF*), representing a shared domain featured in PpsD and PpsE, phenotypically displaying increased antibiotic tolerance to low-level rifampicin. This study highlights the power of using controlled laboratory studies to uncover minority variants in populations of *M. tuberculosis*. These outcomes will lead to improved diagnosis of antibiotic resistance emergence in TB, to optimize management and treatment of TB infections, and ultimately to minimize patient deaths.

**Impact Statement**

With the emergence of antibiotic resistance in the world’s deadliest disease, tuberculosis, studies investigating the mechanisms of resistance in *Mycobacterium tuberculosis* are essential to minimize patient deaths. However, due to the difficulty of lab-based studies on *M. tuberculosis*, most studies to date focus on analysing resistant strains arising in the clinic and identifying their primary mutations. To date, no controlled laboratory evolution utilizing multi-step increasing exposure to antibiotics, combined with population-level sequencing, has been used to uncover progressive acquisition of resistance determinants in whole populations. In this study, we applied a novel directed evolution approach that induces resistance to the first- and last-line antibiotics, rifampicin and linezolid, in populations of *M. tuberculosis* and used whole-genome sequencing to monitor development of resistance mutations across the population over time. We uncover novel mutations not yet linked to antimicrobial resistance, often found at low frequency in the resistant population and would otherwise go undetected in traditional resistance studies of *M. tuberculosis*. Additionally, we demonstrate that identified gene(s) (*ppsD*/*E*) confer low-level rifampicin resistance in an additional species – highlighting the importance of this method to studying complex diseases like TB, but also broader implications for other critical antibiotic-resistant bacterial species.

## Data Summary

**Fig. 1.**
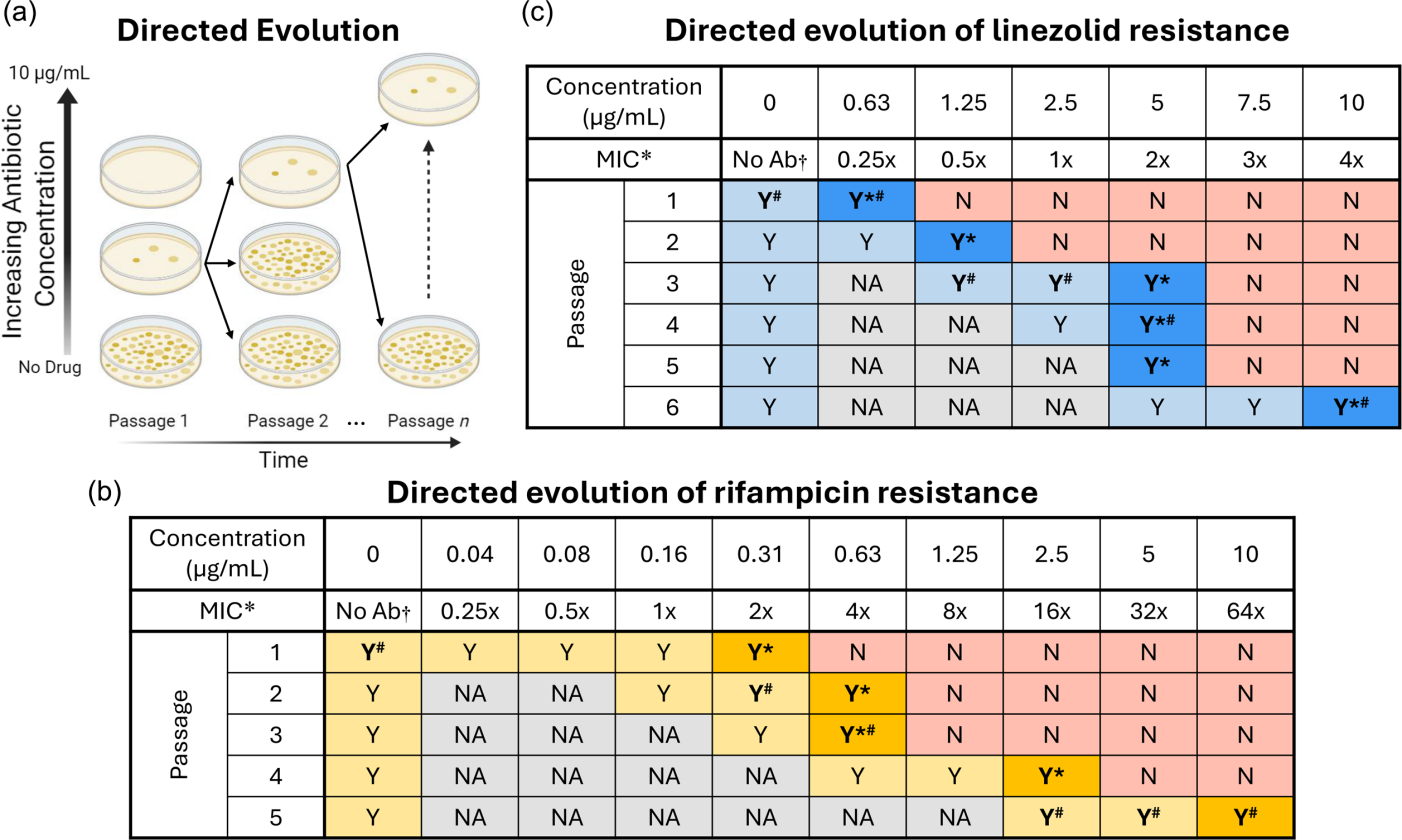
Directed evolution of antibiotic resistance in *M. tuberculosis* H37Rv. (a) Workflow of directed evolution to evolve rifampicin- or linezolid-resistant *M. tuberculosis* populations. Evolved resistant populations for (b) rifampicin, with an estimated 64-fold MIC increase by the 5th round of selection, and (c) linezolid, with an estimated four-fold MIC increase by the 6th round of selection. * Indicates MIC relative to those determined in liquid microdilution in accordance with EUCAST methodology, determining MIC as 0.16 µg ml^−1^ for rifampicin and 2.5 µg ml^−1^ for linezolid. † No antibiotic (No Ab). Darker colour fill indicates highest concentration of antibiotic supporting growth for each passage. ‘Y’ indicates growth, ‘N’ indicates no growth and ‘NA’ indicates not analysed. Bold face type ‘Y’ indicates samples used in subsequent experimental steps, where colonies from ‘Y*’ were sequentially transferred to plates with higher antibiotic concentrations in subsequent selection rounds, and colonies from ‘Y#’ were used for WGS.

**Fig. 2.**
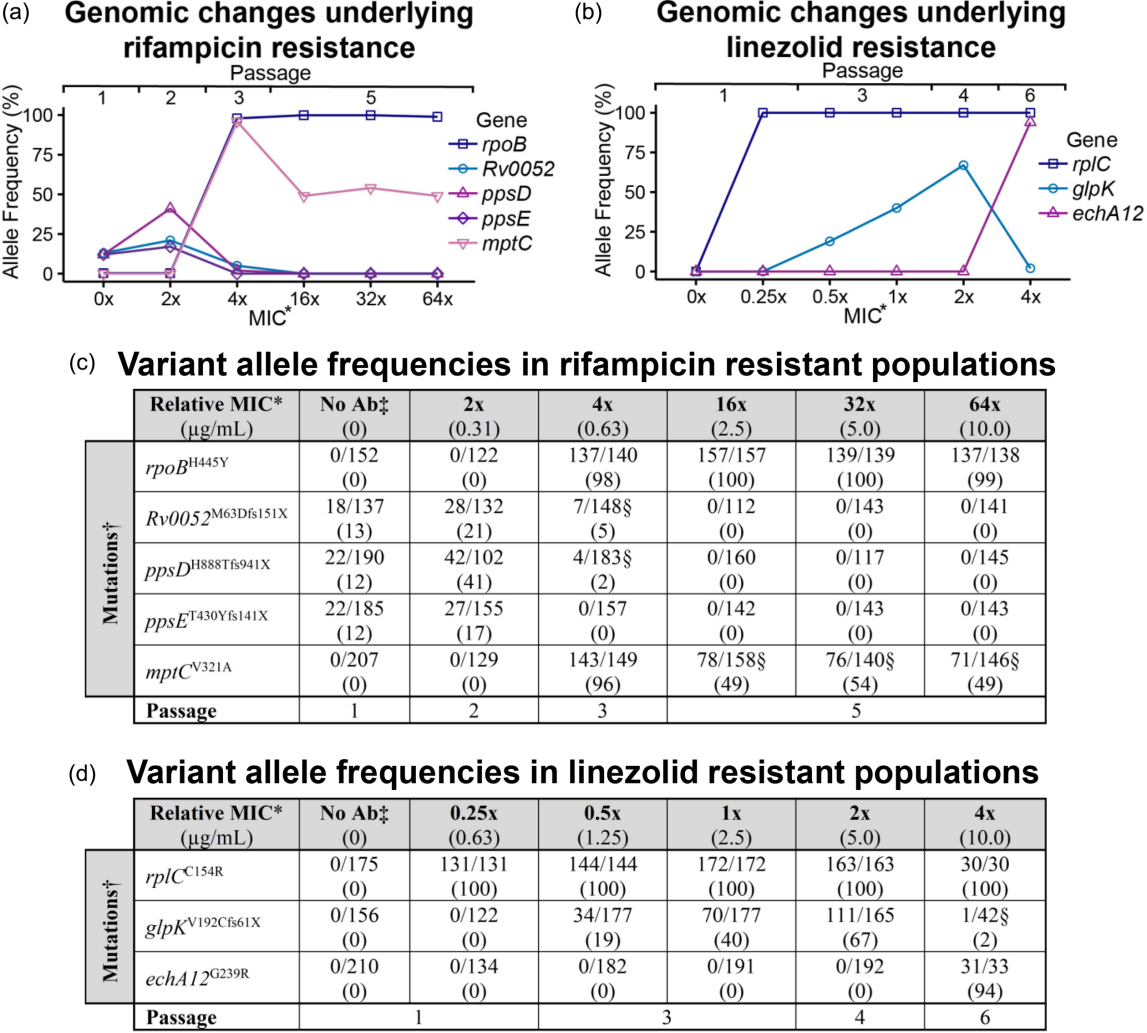
Variant AFs of identified genes within *M. tuberculosis* populations, giving stepwise resistance phenotypes of increasing MIC. Visualized acquisition of variants (%) to (a) rifampicin and (b) linezolid. Exact number of reads with the variant present out of the total reads, with percentage below (%), for (c) rifampicin- and (d) linezolid-resistant populations as identified by the variant calling pipeline used in this study (see methods). * MIC represents the relative MIC to those determined in liquid media. Numbers in brackets represent the concentration (μg ml^−1^). † Mutations in superscript represent changes in the amino acid sequence. ‘X’ indicates a stop codon and ‘fs’ indicates a frameshift mutation. ‡ No antibiotic (No Ab). § Indicates presence determined by manual assessment of read outputs, but not detected by variant calling pipelines.

Population-level sequencing data of the TB-resistant passages (Figs 1 and 2) can be found in ENA under the study accession number: PRJEB82621. Individual accessions are as follows: ERR13962878 ERR13962879, ERR13962880, ERR13962881, ERR13962882, ERR13962883, ERR13962884, ERR13962885, ERR13962886, ERR13962887 and ERR13962888. Data, scripts and bioinformatics pipeline used in the analysis of genomic variants and resistance phenotypes can be accessed via GitHub at https://github.com/jazmia/Variant_Analysis. The authors confirm all supporting data, code and protocols have been provided within the article or through supplementary data files.

## Introduction

The emergence of antibiotic resistance in *Mycobacterium tuberculosis*, the bacterium that causes tuberculosis (TB), poses one of the greatest public health threats in history. The current TB pandemic, where active and latent infections of *M. tuberculosis* are estimated to occur in a quarter of the world’s population and cause ~1.5 million deaths each year, represents the world’s leading cause of death from a single infectious agent [1,2]. Further, this deadly disease is becoming increasingly difficult to treat owing to the global rise of antibiotic resistance [1,3]. Standard treatment of TB involves a 6-month course of a combination of three to four anti-TB drugs, yet even this involvement regimen only yields an 85% cure rate [2]. Lengthy treatments are required due to the ability of TB to enter a dormant (non-replicating) state where, alongside other innate resistance mechanisms, *M. tuberculosis* is able to resist antibiotics [4]. However, prolonged treatments provide continuous drug exposure and increase the risk of patient non-adherence, thereby creating a perfect storm of selective pressures for antibiotic-resistant *M. tuberculosis* to emerge [5].

*M. tuberculosis* can develop resistance to a wide spectrum of antibiotics, ranging from single resistance to first-line drugs, to extensively drug-resistant against multiple and last-line therapies [2]. Unlike other critical priority pathogens like the *Enterobacteriaceae*, which horizontally exchange resistance determinants, resistance in *M. tuberculosis* is primarily mediated via single-point mutations or homologous recombination under antibiotic selective pressure [5,6]. Due to the *de novo* nature of resistance mutation development in *M. tuberculosis*, populations can be heterogeneous, comprised of sensitive and resistant subpopulations that are otherwise isogenic [7]. Multiple mutations may be observed in a single population as resistance mutations can come at a fitness cost, often requiring compensatory mutations to maintain fitness, while additional mutations may boost resistance levels [8]. Thus, understanding this complex interplay among selective pressures, population resistance acquisition and fitness is crucial for addressing *M. tuberculosis* resistance and may uncover novel or low-level compensatory mutations supporting overall resistance [1,2].

Rifampicin-resistant *M. tuberculosis* has recently been classified by the World Health Organization (WHO) as one of only four ‘critical’ priority pathogens in urgent need of new antimicrobials [1,2]. Rifampicin (RIF) is the most potent and commonly used first-line drug to treat TB [2,9]. It kills *M. tuberculosis* by binding to the *β*-subunit of the RNA polymerase, inhibiting transcription [9,10]. However, in 2023, ~400,000 TB cases were multidrug-resistant or rifampicin-resistant, rendering treatment ineffective [2]. The vast majority of rifampicin resistance is conferred by SNPs in *rpoB* (~95%), altering the target *β*-subunit of the RNA polymerase to prevent binding [6,11, 12]. Mutations in *rpoA* and *rpoC*, which encode the RNA polymerase *α*- and *β*’-subunits, respectively, are observed to co-occur in approximately one-third of *rpoB* mutants as compensatory mutations [12,13].

Linezolid (LZD) is an oxazolidinone antibiotic that has been repurposed for the treatment of TB and is currently recommended by WHO as a last-line treatment to treat multidrug-resistant/rifampicin-resistant infections [2,14]. LZD kills *M. tuberculosis* by binding to the 23S rRNA of the 50S ribosomal subunit, thereby inhibiting translation [14]. The primary genes associated with linezolid resistance are *rplC* and *rrl*, which encode the ribosomal protein L3 and the 23S rRNA, respectively, and SNPs in these genes prevent antibiotic binding to these targets [6,14]. *rplC* and *rrl* are estimated to represent 36.8 and 10.5% of linezolid resistance cases, respectively. Therefore, there is a need to expand the extremely limited catalogue of linezolid resistance mutations [12,15, 16].

Owing to the global importance of ensuring viable TB treatment options for this prevalent and severe infectious disease, we assessed the spectrum of mutations that arise at a population level, as *M. tuberculosis* evolves resistance to first- and last-line anti-TB drugs. Most studies to date focus on analysing resistant strains arising in the clinic and identifying their primary mutations (e.g. *rpoB*, *rplC*), although a handful of studies have used a comprehensive metadata approach or generated resistant isolates via single-step exposure to uncover novel mutations [17,18]. To date, no controlled laboratory evolution utilizing multi-step progressive exposure to increasing concentrations of antibiotics, combined with population-level sequencing approaches, has been used to uncover low-level resistance variants. To address this, we performed the first controlled laboratory evolution of *M. tuberculosis* communities under stepwise exposure to rifampicin and linezolid, coupled with population-level whole-genome sequencing (WGS), adapting previously developed methodologies to *M. tuberculosis* [19]. From this, we identified the drivers of resistance development in *M. tuberculosis* in a controlled, stepwise manner, assessing genetic variability within populations over time. The outcomes of this study will inform future treatment strategies to minimize the development of resistance and ensure successful treatment, and guide development of much-needed new TB drugs and combinations.

## Methods

### *M. tuberculosis* strain and minimum inhibitory concentration

*M. tuberculosis* H37Rv (ATCC 27294) was used for this study. The minimum inhibitory concentration (MIC) for rifampicin (Sigma-Aldrich) and linezolid (MedChemExpress) was determined across a range of 0.039–5 µg ml^−1^, in line with the standardized EUCAST methodology used in clinical settings [20]. Antibiotic dilutions were prepared in Middlebrook 7H9 (M7H9, BD Difco) supplemented with 10% oleic acid-albumin-dextrose-catalase (BD Difco) and 0.2% glycerol. Briefly, 100 µl of antibiotic dilution and 100 µl of inoculum were prepared in a U-shaped 96-well polystyrene microtitre plate (Corning) with a final inoculum of 10^5^ c.f.u. ml^−1^. Plates were incubated at 36±1 °C for 7 days. MICs were determined using the resazurin microtitre assay [21], which relies on an oxidation-reduction indicator. After the incubation period, 30 µl of resazurin solution was added to each well and incubated overnight at 37 °C. Colour change was then observed, with a shift from blue to pink indicating bacterial growth due to the reduction of resazurin. MICs were defined as the lowest concentration of the drug that prevented this colour shift, indicating inhibition of bacterial growth.

### Directed evolution of antibiotic-resistant *M. tuberculosis* mutants

Our previously described directed evolution methodologies [19] were adapted for use on *M. tuberculosis* H37Rv ATCC 27294. *M. tuberculosis* H37Rv mutants were selected through culturing 10 µl of a 1 ml M7H9 liquid culture (10^6^ cells) on antibiotic-supplemented M7H9 agar plates, in triplicate. Plates were supplemented with stepwise increases in antibiotic concentrations, starting at 0.25× their MICs. When full population growth was achieved (~2 months per passage), the populations were re-passaged, with mutants collected from the highest concentration plate, resuspended in 1 ml of M7H9 broth, and used to inoculate new plates with a stepwise antibiotic gradient – thereby ‘training’ the population to increase resistance. *M. tuberculosis* was cultured in non-selective media as a growth control for directed evolution experiments. For both antibiotics, concentrations started at 0.25× MIC with two-fold increases, reaching 64× MIC for rifampicin and 4× MIC for linezolid (with one 1.5-fold increase). Additionally, mutants from each passage were isolated, purified and stored in 15% glycerol at −80 °C for further investigation. Although we report resistance phenotypes on solid media relative to MIC determined in liquid media to align with clinical MICs determined by standardized EUCAST methodologies, we also acknowledge that there can be variations between liquid and agar MIC values [20,22].

### DNA extraction, library preparation and WGS

Isolates were sub-cultured on Löwenstein–Jensen medium and incubated at 37 °C for 4 weeks. Several colonies of *M. tuberculosis* were collected for DNA extraction following the cetyltrimethylammonium bromide protocol and resuspended in nuclease-free water [23]. DNA quality was assessed by Nanodrop and gel electrophoresis, and quantity was measured by Nanodrop and Qubit Fluorometer (Table S1, available in the online Supplementary Material). Whole-genome paired-end Illumina sequencing was performed by the Australian Institute for Microbiology and Infection, University of Technology Sydney. DNA was prepped using the Nextera Flex kit before sequencing on a NovaSeq 2×150 PE sequencer, generating >2 million reads to >40× coverage (Table S1). Individual sequencing reads were deposited in the European Nucleotide Archive under accession number PRJEB82621 (https://www.ebi.ac.uk/ena/browser/view/PRJEB82621).

### Identification of genomic variants and their function

Clinical pipelines, including the WHO TB resistance database [12], comprehensive antibiotic resistance database (CARD) TB mutations [24], Mykrobe [25] v0.10.0, TBProfiler [26,27] v6.2.2, TBPortals Genomic Analysis Pipeline [28], DrPRG [29] v0.1.1 and tbtAMR [30] v1.0.3, were used as an initial assessment of known resistance alleles. However, for the detection of novel resistance alleles, genomic variants were detected as previously described [19] by comparing aligned sequence reads of resistant isolates to the parental strain. Sequencing data were assessed for quality using FastQC [31] v0.12.1 and trimmed accordingly using Trimmomatic [32] v0.39 (Table S1). Reads for the parental strain were aligned to the *M. tuberculosis* H37Rv reference genome (GenBank: CP110619), while reads for each mutant strain were aligned to the consensus genome sequence of the parental strain. Reads were aligned using BWA [33] v0.7.17 (r1188) and consensus genome sequences for each strain were produced using SAMtools [34] v1.19.2. BCFtools [34] v1.19 was then used to identify single-nucleotide variants and short insertions/deletions (indels) in each mutant strain from their mapped reads. Mutations were also identified with Snippy [35] v4.6.0 pipeline using the trimmed reads and the H37Rv reference genome. Additionally, all mutations identified were assessed for accuracy by visualizing BAM files in IGV [36] v2.16.0 for rifampicin- and linezolid-resistant populations (Figs S1 and S2, respectively). ORFs in consensus genomes for the parental and mutant strains were annotated using Prokka [37] v1.14.6, with mutations that induced a change in the amino acid sequence considered mutations of interest in this study. Proteins of interest were confirmed using blastp v2.16.0 against the non-redundant protein sequence database, with hypothetical proteins further analysed for potential function using BioCyc [38] v4 and InterPro [39]. The existence of specific mutations in previously published *M. tuberculosis* sequences was searched against the blastp non-redundant protein sequence database, blastn v2.17.0 core nucleotide database and unassembled FASTA reads from published clinical *M. tuberculosis* isolates deposited on the Sequence Read Archive. Protein domains were predicted by InterPro and the relevant domain containing the mutation was aligned with equivalent domains from published whole-genome sequences using muscle [40], and a phylogenetic maximum likelihood tree was constructed by mega11 [41] using complete sequences with 100 bootstrap replications.

### Growth curves and membrane permeability assays

*M. tuberculosis* genes associated with rifampicin and linezolid resistance were assessed for resistance phenotypes using single-gene knockout mutants of *Escherichia coli*. Methods are described in further detail in the Supplementary Methods. Briefly, *E. coli* K12 BW25113 Δ*fabF* (Table S2), an identified ortholog to *M. tuberculosis ppsD*/*E*, was assessed for resistance phenotypes using the broth microdilution method [42]. The MIC was determined across a range of 2.5–60 µg ml^−1^ of rifampicin, while growth was assessed over a finer range of 2–20 µg ml^−1^ of rifampicin.

The possible mechanism underlying the tolerance phenotype of Δ*fabF* was further investigated by assessing relative membrane permeability through flow cytometric assessment of fluorescence, corresponding to SYTOX Green (Thermofisher, S7020) uptake, using a BD FACSymphony^™^ A1 Cell Analyzer, conducted by the Australian Genome Foundry (Macquarie University, Sydney, Australia). In total, ~50,000 single-cell events per replicate were recorded from triplicate cultures of *E. coli* K12 WT and Δ*fabF* with treated samples cultured in 8 µg ml^−1^ rifampicin for 18 hrs, relative to a dead cell control (Fig. S3).

## Results and discussion

### Directed evolution of rifampicin- and linezolid-resistant *M. tuberculosis* populations

To assess the acquisition of rifampicin and linezolid resistances in *M. tuberculosis*, we conducted directed evolution experiments over six successive passages (each lasting ~8 weeks) using the antibiotic-sensitive strain H37Rv (the parental strain) with no prior exposure to either drug (Fig. 1). In the first round of selection on rifampicin (passage 1), the strain H37Rv was able to form colonies on plates containing up to 2× the MIC (Fig. 1b). After four additional rounds of successive re-passaging with increasing rifampicin concentrations (over the time period of ~1 year), *M. tuberculosis* was able to grow on plates containing up to 64× the MIC (Fig. 1b). This pattern of growth suggests that *M. tuberculosis* populations can readily evolve rifampicin resistance in the laboratory using this directed evolution methodology [19].

In contrast, during the first round of selection on linezolid, the parental strain showed population growth only on plates containing sub-inhibitory concentrations (up to 0.25× the MIC). After five additional rounds of selection, colonies were able to grow on plates with concentrations up to 4× the MIC (Fig. 1c), indicating that *M. tuberculosis* had evolved to increase linezolid resistance, albeit at a slower rate than rifampicin resistance. These results demonstrate that serial passaging under stepwise increasing antibiotic pressure can select for populations with enhanced rifampicin and linezolid resistance, providing a controlled framework from which we can investigate the genetic basis and evolutionary dynamics of antibiotic resistance in *M. tuberculosis*.

### Identification of mutations in rifampicin- and linezolid-resistant *M. tuberculosis* populations

To identify the genetic determinants conferring the observed stepwise increases in resistance, WGS was performed on the parental strain H37Rv, and five rifampicin-resistant and five linezolid-resistant evolved populations (indicated in Fig. 1, represented by #), with the total number of SNPs identified outlined in Table S3. As expected, our SNP analysis identified mutations with 98–100% allele frequency (AF) in genes commonly implicated in TB clinical resistance, *rpoB* (RIF) and *rplC* (LZD) (Table 1 and Fig. 2a), which are known to prevent rifampicin and linezolid from binding to their respective targets [6,12]. For rifampicin, the mutation in *rpoB* occurred inside the rifampicin resistance-determining region and was observed to arise at 4× MIC (Fig. 2a and c) [12]. For linezolid, mutation in *rplC* was observed in all linezolid-resistant mutants, even those at sub-inhibitory concentrations at full penetrance (100% AF) (Fig. 2b and d). This suggests that linezolid resistance can arise at lower concentrations relative to the MIC, highlighting the potential for rapid and stable selection under subtherapeutic exposure. These resistance mutations could also be detected using all standard clinical variant pipelines for resistance mutation identification in *M. tuberculosis* (CARD TB Mutations [24], TBProfiler [26,27], Mykrobe [25], TBPortals [28], DrPRG [29] and tbtAMR [30], using default parameters; Table S4), except *rplC* for linezolid resistance, which was not detected via Mykrobe or CARD.

**Table 1.** Genomic changes in annotated genes detected for evolved rifampicin and linezolid populations compared to parental *M. tuberculosis* H37Rv. Outline of function and previous association with antibiotic resistance (AbR)

Antibiotic	Gene (locus ID)	Function	MIC*	Mutation (position in gene)	Amino acid change† (position in protein)	Previous links with AbR‡	Reference
**Rifampicin**	*rpoB* (Rv0667)	Transcription	4–64×	C → T (1,333)	H445Y	Confers RIF resistance	[6]
	*Rv0052* (Rv0052)	Transcriptional regulatory protein (putative)	0–2×	GCC → GCCC (186)	M63Dfs151X	None	
	*ppsD* (Rv2934)	Lipid biosynthesis	0–2×	GCCCCCCC → GCCCCCC (2,655)	H888Tfs941X	*ppsA-E* confer low level and boost resistance to pyrazinamide, upregulated in RIF-resistant *rpoB* mutants	[43,44]
	*ppsE* (Rv2935)		0–2×	CGGGGG → CGGGGGG (1,281)	T430Yfs141X		
	*mptC* (Rv2181)	Glycolipid synthesis (putative)	4–64×	T → C (962)	V321A	Upregulated in response to isoniazid and rifampicin	[49,50]
**Linezolid**	*rplC* (Rv0701)	Translation	0.25–4×	T → C (460)	C154R	Confers LZD resistance	[6]
	*glpK* (Rv3696c)	Glycerol metabolism	0.5–2×	GGGGGGGT → GGGGGGGGT (573)	V192Cfs61X	Supports broad antibiotic resistance, unable to confer resistance alone	[53]
	*echA12* (Rv1472)	Cholesterol metabolism (putative)	4×	G → A (715)	G239R	Florfenicol resistance	[54,59]

*Indicates MIC relative to those determined in liquid microdilution in accordance with EUCAST methodology, determining MIC as 0.16 µg ml−1 for rifampicin and 2.5 µg ml−1 for linezolid, where mutation was detected by variant calling pipelines, excluding detection only by visual assessment.

†Amino acid change where ‘X’ indicates a stop codon and ‘fs’ indicates a frameshift mutation.

‡Antibiotic resistance (AbR).

However, in the passages where these core resistance mutations in *rpoB* (passage 3) and *rplC* (passage 1) had been acquired, the respective *M. tuberculosis* populations were only able to grow on plates up to 4× MIC for rifampicin and 0.25× MIC for linezolid, indicating that additional mutations are necessary to gain the further resistance seen in following passages (Figs 1b, c and 2). Our SNP analysis also identified eight mutations in the resistant populations compared to the parental H37Rv strain that were outside of the catalogue of known resistance mutations: five in rifampicin-resistant populations and three in linezolid-resistant populations (Table 1). Additionally, of the clinical TB resistance variant identification pipelines also used, only tbtAMR and TBProfiler identified low-frequency variants, including variants not detected by the focal pipeline used in this study (Tables S4 and S5).

In the rifampicin selection regime, we also detected subpopulations carrying mutations within the genes *Rv0052* (21% AF), predicted to encode a transcriptional regulatory protein, and *ppsD* (41% AF) and *ppsE* (17% AF), encoding related polyketide synthases (Fig. 2a and c) [6,43, 44]. Interestingly, these mutations were already present in the parental strain population (passage 1), albeit at low frequency, indicating standing genetic variation prior to antibiotic exposure (Fig. 2c). Their AFs fluctuate during rifampicin exposure, increasing initially but declining following the emergence of the core *rpoB* mutation. As the *rpoB* mutation appears to arise in a different genetic background, the observed frequency shifts likely reflect changes in the relative abundance of distinct subpopulations rather than reversion within a single lineage. This pattern is consistent with heteroresistance [45] and clonal interference and suggests that these variants may contribute to transient stress adaptation or antibiotic tolerance.

These genes have not previously been identified as rifampicin resistance mutations in the majority of the TB resistance databases, including the WHO TB resistance database [12], TBProfiler, Mykrobe, TBPortals or DrPRG databases. However, CARD indicated that alternative mutations to *ppsD* can confer high-level resistance to pyrazinamide, another TB treatment, with *ppsD*/*E* mutants also been shown to confer low-level resistance to pyrazinamide (Table 1) [44].

In addition to the alleles described above, we detected an *mptC* allele carrying a substitution mutation at high frequency (96% AF) at passage 3 (4× MIC), coinciding with the initial sweep of the population by the *rpoB* H445Y allele (Fig. 2a and c). Interestingly, the *mptC* AF subsequently decreased to ~50% and remained stable at this level under higher rifampicin concentrations, whereas the *rpoB* H445Y allele reached fixation (100%). The co-occurrence of these mutations is noteworthy and may suggest a functional interaction, potentially indicative of a compensatory or adaptive role. However, the *rpoB* H445Y mutation alone has been reported to confer resistance up to 100 µg ml^−1^ of rifampicin [46]. Additionally, the equivalent *rpoB* mutation in *E. coli* (H526Y) has been shown to confer increased fitness under environmental stress compared to the wild-type and other rifampicin resistance mutations, suggesting relatively low fitness costs and therefore a limited requirement for compensatory adaptation [17,47, 48].

The *mptC* gene is predicted to encode an alpha-(1-2)-phosphatidylinositol mannoside mannosyltransferase and has not been previously linked to rifampicin resistance (Table 1), although the functionally related *mptA* gene [49] has been associated with isoniazid resistance [50]. While additional low-frequency mutations were detected by tbtAMR and TBProfiler in *ettA* (2× MIC) and *embB* (L172R at 2× MIC and L172R and S174R at 64×, Tables S4 and S5), the coverage at *ettA* was unknown, while the coverage at *embB* was much lower than that for the rest of the genome (35× compared to >100× coverage), and thus these mutations were not considered for further investigation.

Further increases in linezolid resistance were associated with additional mutations that co-occurred with the *rplC* mutation. Mutations in *glpK*, encoding a glycerol kinase, were observed between 0.5× and 2× the MIC (passages 1–4), initially arising with the acquisition of the core *rplC* mutation (100% AF) and progressively increasing in prevalence (19–67% AF) and also detected by TBProfiler (Fig. 2b and d). A secondary *glpK* mutation (W322R, Tables S4 and S5) was also detected by both tbtAMR and TBProfiler, however, with a frequency <15% and the much higher prevalence of the other *glpK* mutation, it was not considered for further investigation. Although mutations in *glpK* have previously been observed in rifampicin-, isoniazid-, ethambutol-, streptomycin-, pyrazinamide- [51,52], levofloxacin- [12] and moxifloxacin-resistant isolates [53], they are considered not to be associated with resistance and have not been observed in linezolid-resistant isolates in the WHO TB resistance database [12]. However, *glpK* knockout mutants have also been demonstrated to show drug tolerance to rifampicin, isoniazid and moxifloxacin [53]. At the highest level of linezolid resistance (4× MIC, passage 6), *glpK* mutation prevalence in the population drastically decreased to 2% AF and was replaced by a mutation in the completely novel resistance gene *echA12* (94% AF), encoding a probable enoyl-CoA hydratase (Fig. 2b and d) [54]. Both mutations appear to have an additive effect to complement the core *rplC* mutation, with *echA12* taking over the role of *glpK* at the highest level of linezolid resistance assessed.

Our variant calling analysis has revealed several potential new mutations and genes associated with rifampicin and linezolid resistance in *M. tuberculosis*, many of which co-occur with common resistance in *M. tuberculosis* populations, namely *rpoB* and *rplC*. Although tbtAMR and TBProfiler appear to be the only clinical variant calling pipelines that demonstrated the capacity to identify minority variants in *M. tuberculosis* populations, demonstrating the capacity [*ettA* and *embB* (RIF) and *glpK* (LZD) mutations]. As demonstrated by the inability of *M. tuberculosis* populations with core resistance mutations being unable to gain further resistance without the acquisition of additional mutations, most of which did not occur at full penetrance, the detection of minority variants is an important aspect of understanding resistance in *M. tuberculosis*. These findings highlight the limitations of standard TB resistance variant pipelines and even widely used generalized variant calling pipelines, including Snippy, for detection of novel, intrinsic or low-level mutations [29]. While only the general variant calling pipelines used in this study were able to identify novel mutations (Table S4), alterations to more user-friendly, existing pipelines with capacity for low-frequency variant identification, such as tbtAMR and TBProfiler, to output high-confidence resistance and resistance-associated mutations may inform the clinical management of resistant TB cases. Accruing more data on variants associated with resistance can compile those associated with resistance and also provide a priority list of those that should be followed up with functional validation to become high-confidence. This will support continuously tracking novel resistance mechanisms in *M. tuberculosis*, informing clinical management of resistant TB cases. To further strengthen that the variants identified in this study are due to antibiotic selection, sequencing could be conducted on an untreated passaging control to eliminate variants that can occur during the multiple passaging due to time, ageing, prolonged *in vitro* culture or genetic drift. Although this was not done due to initial experimental design, five out of the six novel mutations identified have previous links to antibiotic resistance (Table 1). Additionally, sequencing additional colonies at each passage may have improved the representation of variants identified through attempting to capture more variants or differing frequencies. However, the additional samples require highly restricted resources and a significant time investment (>1 year), often not generating enough resistant *M. tuberculosis* colonies at each passage to sequence and use for further passages, so it was not feasible for the current investigation.

### Novel rifampicin- and linezolid-resistant mutations

While core resistance mutations, *rpoB* (RIF) and *rplC* (LZD), were identified in most samples (Fig. 2), they alone do not explain the continued stepwise increases in resistance over each passage. The community-level sequencing approach revealed six additional mutations, with five observed as minority subpopulations, that had not been previously linked to rifampicin or linezolid clinical resistance [12]. This allowed the identification of four novel rifampicin resistance mutations (*Rv0052*, *ppsD*, *ppsE*, *mptC*) and two mutations with a novel association to linezolid resistance (*glpK* and *echA12*). A similar stepwise fixation of spontaneous mutations in distinct genes occurs for resistance to bedaquiline in *M. tuberculosis*, both *in vitro* [8] and within TB patients [55]. Notably, pre-existing resistance to bedaquiline has been associated with TB treatment failure [55]. Substantial increases in the MIC of bedaquiline are thought to arise initially from mutations in the efflux pump regulator gene *Rv0678*, followed by subsequent mutations in *atpE*, the bedaquiline target gene, during sequential passages under bedaquiline selection pressure [8].

Among the mutations in the current study, only the *glpK* mutation is represented in the National Center for Biotechnology Information (NCBI) core nucleotide database (Table S6). However, further investigation of unassembled WGS FASTA reads from clinical, RIF-profiled, TB isolates available in the NCBI database revealed that the *ppsD*, *glpK* and *mptC* resistance mutations identified in this study could also be observed in some rifampicin-resistant clinical isolates (Table 2). These reads may represent mutant subpopulations but remain undetected in a clinical context using standard resistance detection analysis due to low penetrance. Furthermore, when comparing the variability within rifampicin resistance in a strain set of published, clinical *M. tuberculosis*, clustering of the consensus protein domains from strains with a resistance phenotype does not occur for any protein except RpoB, which clustered into two major clades (Fig. S4). This mutation distribution in consensus sequences, for the proteins other than RpoB, may suggest that these mutations have low penetrance and are consequently lost in the consensus sequence or are likely to occur sporadically and are not obviously attributed to a single event followed by clonal expansion or a combination of both hypotheses. There is an insufficient number of WGS data of linezolid-resistant isolates to perform a similar analysis due to a paucity in phenotypic screening for linezolid resistance [56].

**Table 2.** Unassembled FASTA reads of published clinical *M. tuberculosis* containing rifampicin- or linezolid-associated mutations detected in evolved population H37Rv. Proportion of sequencing reads (%) with mutations compared to total reads of each sequence. MDR=resistant to rifampicin and isoniazid. Pre-XDR=MDR plus resistance to a fluoroquinolone

Raw read accession	Strain	Gene	Mutation (position in gene)	Amino acid change* (position in protein)	Read % with mutation	Resistance phenotype
SRR10828189	25474	*glpK*	GGGGGGGT → GGGGGGGGT (573)	V192Cfs61X	10.81	pre-XDR
SRR10828193	29366	*glpK*	GGGGGGGT → GGGGGGGGT (573)	V192Cfs61X	2.33	pre-XDR
SRR10828621	37032	*ppsD*	GCCCCCCC → GCCCCCC (2,655)	H888Tfs941X	0.52	pre-XDR
SRR10828641	40543	*rplC*	T → C (460)	C154R	0.41	pre-XDR
		*glpK*	GGGGGGGT → GGGGGGGGT (573)	V192Cfs61X	1.2	
SRR11662278	40868	*rpoB*	C → T (1,333)	H445Y	100	pre-XDR
		*ppsD*	GCCCCCCC → GCCCCCC (2,655)	H888Tfs941X	1.03	
		*mptC*	T → C (962)	V321A	0.44	
SRR8186761	TCDC3	*ppsD*	GCCCCCCC → GCCCCCC (2,655)	H888Tfs941X	0.36	MDR
		*glpK*	GGGGGGGT → GGGGGGGGT (573)	V192Cfs61X	0.83	

*Amino acid change where ‘X’ indicates a stop codon and ‘fs’ indicates a frameshift mutation.

While there is very limited characterization of *Rv0052*, the amino acid sequence of Rv0052 was matched with 100% identity and query coverage to a DJ-1/PfpI family protein (WP_003912418.1) and indicated to be involved with regulation of DNA-templated transcription in Uniport (I6Y6S3). This mutation is also present at a low frequency in the parental H37Rv strain but not present in any higher-level resistance mutants, suggesting that the mutation is only effective in lower-level concentrations and is selected against when higher levels of rifampicin are present. Further experimentation will be required to define the exact role of this mutation in rifampicin resistance.

Both *ppsD* and *ppsE* encode subunits of a type I polyketide synthase complex involved in the biosynthesis of phthiocerol dimycocerosate, which are lipids located in the outer mycobacterial cell wall, implicated in virulence and required for *in vivo* growth [43,44]. Mutants with low-level rifampicin resistance have low-frequency *ppsD* mutations, with a restorative mutation AF of 41%, while the parental strain sequence structure, which results in a truncated protein that annotates as two separate *ppsD* genes, has an AF of 59% in these low-level rifampicin-resistant mutants. The two separate PpsD proteins lack the polyketide dehydratase domain, suggesting a loss of function. Similarly, the *ppsE* mutation, also observed at this level of resistance, results in a truncated protein, with the loss of the acyl-synthase domain likely leading to loss of function. The restorative mutation of *ppsD* is consistent with the upregulation of polyketide synthase genes as a known mechanism to compensate for *rpoB* mutation [43]. Notably, frameshift mutations in *ppsA-E* have been linked to low-level pyrazinamide resistance and found to boost pyrazinamide resistance conferred by *panD* in *M. tuberculosis* [44]. However, like *Rv0052*, the *ppsD* mutation is present in the parental H37Rv, while higher resistance mutants do not feature this mutation.

The *mptC* gene encodes an enzyme involved in the synthesis of mannose-capped lipoarabinomannan, a glycolipid found in the cell wall of *M. tuberculosis*, where it functions as a virulence factor, supports survival and helps evade host defence mechanisms [49,50]. *mptC* is upregulated in rifampicin- and isoniazid-resistant *M. tuberculosis*, suggesting that *mptC* protects the mycobacterial cell [50]. It is uncertain what effect the conservative missense mutation has on MptC functionality, although the knockout of *mptC* is predicted to provide a growth advantage in 7H9 and 7H10 with 10% oleic acid-albumin-dextrose-catalase media [57]. This growth advantage, combined with the high AF relative to the other mutations associated with RIF resistance (Fig. 2a and c), could support a hypothesis that *mptC* mutations in particular may improve fitness, which is impaired by *rpoB* mutation [58]. However, further investigation is required to validate *mptC* in the context of rifampicin resistance.

Additionally, low-frequency mutations found in linezolid-resistant mutants include *glpK* and *echA12*. *glpK* mutations were observed in linezolid-resistant strains between 0.5× and 2× MIC, increasing in frequency as antibiotic concentration increased (Fig. 2b and d). This exact frameshift mutation in *glpK* has previously been detected in clinical *M. tuberculosis* isolates (Table S6), including in isolates from *in vivo* murine infection models treated with moxifloxacin [53]. Consistent with our findings, these *glpK* mutants were phase variants, associated with an antibiotic-tolerant phenotype. GlpK is a glycerol kinase, so *glpK* frameshift mutants are likely unable to metabolize glycerol and therefore activate a stress response, supporting antibiotic resistance. This previous study also assessed single knockout mutants of *M. tuberculosis* H37Rv with a deletion of *glpK* and demonstrated increased percentage growth in low levels of rifampicin, but they were also found to have the same MIC as the wild-type, indicating that *glpK* mutations are unable to confer high levels of resistance without additional mutations [53].

The *echA12* is predicted to encode an enoyl-CoA hydratase, by sequence similarity, with other *M. tuberculosis* enoyl-CoA hydratases involved in lipid membrane metabolism and cholesterol metabolism [54,59, 60]. While not previously observed in linezolid resistance, laboratory-evolved florfenicol-resistant *Mycobacterium smegmatis* mutants feature the same SNP in *echA12* (G239R) [60]. The mutated *echA12 is* subsequently overexpressed, resulting in increased resistance to florfenicol [60]. Despite the low sequencing coverage, the high frequency of this *echA12* mutation suggests it is a high-level resistance mutation. Additionally, mutants possessing this mutation have a large decrease in the prevalence of the *glpK* mutation seen in prior stages of resistance, indicating that at this stage of resistance, *echA12* supersedes *glpK* in providing a fitness benefit.

Out of the eight resistance mutations identified in this study, half were frameshift mutations (*Rv0052*, *ppsD*/*E*, *glpK*) occurring in mononucleotide repeats, a known mechanism of phase variation in *M. tuberculosis* [53]. Phase variation is an adaptive mechanism that allows genes to be rapidly switched ‘on’ and ‘off’ by genotypic changes [53]. This includes the insertions/deletions observed in this study in mid-level resistant populations, indicating that *M. tuberculosis* is heavily reliant on this adaptive mechanism to mediate the acquisition of higher levels of resistance. The minority variants identified in this study also appear to share commonalities within their respective antibiotic resistance groupings. With the exception of *Rv0052*, rifampicin mutations observed in *ppsD*/*ppsE* and *mptC* are all genes linked to mycobacterial cell wall components [61]. On the other hand, genes linked to linezolid, *glpK* and *echA12*, are both linked to alternative carbon source metabolism [53,59]. These commonalities represent distinct antibiotic-specific alternative resistance strategies observed to be employed by heteroresistant *M. tuberculosis* populations in this study.

While we have been unable to perform a direct functional validation of each novel mutation, stepwise tracking allows the connection of phenotypic resistance progression with genotypic evolution [62]. Therefore, the temporal association of these novel mutations with increasing MIC supports a likely role in resistance enhancement. Thus, providing a short list of key genes associated with resistance for further functional validation.

### Novel rifampicin tolerance conferred by *ppsD*/*E* ortholog *fabF*

Having identified several new genes, whose mutations are potentially associated with rifampicin and linezolid resistance in *M. tuberculosis*, we next assessed whether disruption to these genes would allow cells to tolerate antibiotics. Owing to laboratory restrictions and the difficulty surrounding genetically modifying *M. tuberculosis*, we opted to conduct a preliminary test using mutants of gene orthologs in *E. coli*. *E. coli* was chosen as a model strain due to its ease of genetic manipulation and previous use in anti-mycobacterial drug discovery, due to the similar structure of its outer membrane to *Mycobacterium*, despite differences in membrane composition [18,63]. Of the six *M. tuberculosis* genes we had identified as potentially associated with rifampicin or linezolid resistance (not in the WHO TB resistance database), we further investigated *ppsD*/*E* due to previous links with pyrazinamide resistance. Further, both ppsD/E displayed a disruptive frameshift mutation that can be assessed with a knockout mutant and had a conserved, non-essential ortholog in *E. coli*. For both *ppsD*/*ppsE*, a single ortholog, with a conserved domain, *fabF*, was identified as an appropriate *E. coli* K12 ortholog. *fabF* and *ppsD*/*E* are similarly involved in the elongation of fatty acyl chains during lipid and type II fatty acid biosynthesis, crucial to the production of cell membranes for the respective hosts [61,64] (Table S7). A targeted transposon insertion mutant was used for further investigation of its rifampicin resistance phenotype (Table S2).

**Fig. 3.**
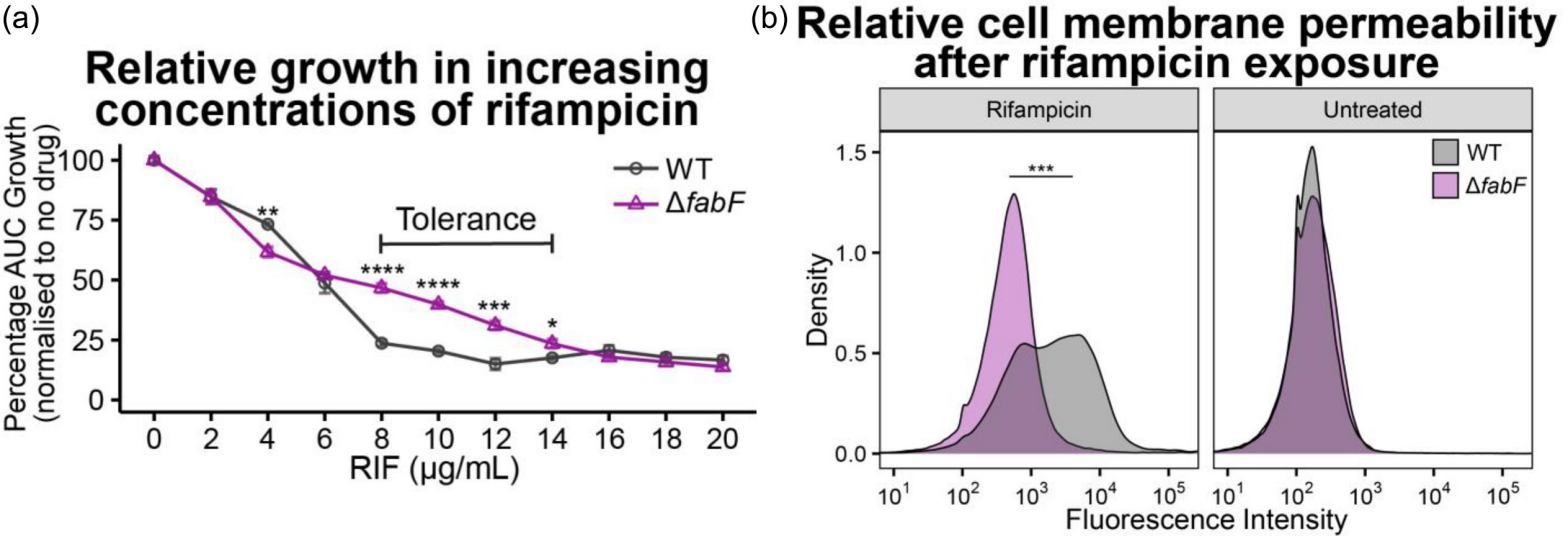
Characteristics of *E. coli* K12 and its Δ*fabF* mutant in response to rifampicin. (a) Growth dynamics (mean±se) of wild-type *E. coli* K12 and Δ*fabF* mutant across 2–20 µg ml^−1^ of rifampicin, with improved growth in the Δ*fabF* mutant between 8 and 14 µg ml^−1^ of rifampicin. (b) Membrane permeability of wild-type *E. coli* K12 and Δ*fabF* mutant, where the Δ*fabF* mutant shows decreased membrane permeability relative to the wild-type after rifampicin exposure (18 h growth in 8 µg ml^−1^). Differences between wild-type and Δ*fabF* mutant were statistically analysed using a t-test, where median fluorescence intensity difference was used for fluorescence data. *P* value: * *P* < 0.05, ** *P* < 0.01, *** *P* < 0.001 and **** *P* < 0.0001.

Using MIC and concentration gradient growth assays, we observed that Δ*fabF* displayed increased growth between the range 8 and 14 µg ml^−1^, as compared to the wild-type (Fig. 3a and Table S8). This indicates that mutations in *fabF* do result in an increased rifampicin tolerance phenotype but not a high-level resistance phenotype per se and may explain why these mutations were only observed in the lower concentrations of rifampicin (Fig. 2a and c). Due to the role of *fabF* in the production of cell membrane lipids, to investigate the mechanism by which the Δ*fabF* mutant mediates this rifampicin tolerance, we performed cell membrane permeabilization assays using SYTOX green uptake as assessed by FACS, on the mutant and wild-type. After treatment with 8 µg ml^−1^ rifampicin, we observed a significant decrease in the permeability of the WT compared to the mutant, but no change was observed without rifampicin treatment (Fig. 3b). Thus, this could suggest that Δ*fabF* mutants were more tolerant to rifampicin exposure due to a less permeable membrane than the wild-type. Surprisingly, rifampicin tolerance mediated by *fabF* has not been reported in *E. coli*, despite being studied thoroughly [65,66]. Given that *fabF* is an *E. coli* ortholog of *ppsD*/*ppsE* in *M. tuberculosis*, with *ppsD*/*E* similarly involved in lipid biosynthesis for the cell membrane [43], these results suggest that *ppsD* and/or *ppsE* may also play a role in rifampicin tolerance in *M. tuberculosis*; however, this still requires further functional validation in an *M. tuberculosis* strain.

Rifampicin tolerance was observed in *E. coli* Δ*fabF* by both improved growth in the Δ*fabF* mutant between 8 and 14 µg ml^−1^ of rifampicin and decreased permeabilization following rifampicin exposure (Fig. 3b). We propose that rifampicin entry into the cell is limited by these mutations that alter the fatty acid composition of the cell membrane. Reduced efficiency in long-chain (>12C atoms) fatty acid elongation decreases the uptake of rifampicin [67,68] and other lipophilic antibiotics [68] into cells. This can confer resistance to lipophilic antibiotics; however, it may also confer a slight reduction of fitness, which is why the native gene is retained and upregulated in *rpoB* mutants [43].

Due to the complexity of *M. tuberculosis* cell envelopes, this *E. coli* model has some limitations. Despite the analogous involvement in fatty acid elongation of PpsD and PpsE in *M. tuberculosis* and FabF in *E. coli*, these enzymes share 26.42 and 29.97% amino acid identity. This limited similarity is due to the entirety of FabF (413AA), representing a ketosynthase family 3 domain profile that is only one of the domains featured in PpsD/E (1827AA and 1488AA) (Table S7). Differences in protein structure, and the requirements for both PpsD and PpsE in *M. tuberculosis*, are likely due to membrane differences between *M. tuberculosis* and *E. coli*. Although limited in amino acid sequence homology, Δ*fabF* phenotypic results are comparable to *ppsD* and *ppsE* mutations in *M. tuberculosis*. Additionally, with their role in cell membrane production, type II fatty acid biosynthesis pathway is of key interest for drug targets [69]. Thus, *ppsD* and *ppsE* may represent potential drug targets for *M. tuberculosis*, in particular due to the necessity of full function for higher levels of resistance [43]. Further, the observed Δ*fabF* tolerance phenotype in *E. coli* indicates that modulation of the cell membrane composition is an important antibiotic tolerance strategy conserved across species, highlighting the broader implications of finding and testing low-level resistance from population-level sequencing in multiple species.

### Implications for TB therapeutics and conclusions

Due to rifampicin and linezolid targeting consecutive steps in protein synthesis, they have been investigated for combination therapies. These studies demonstrate the bacteriostatic effect of these combined antibiotics on rifampicin susceptible isolates, which could prevent the emergence of rifampicin resistance [10,70]. However, there is the potential for rifampicin- and linezolid-resistant mutants arising from combinatorial therapy, in addition to linezolid-resistant mutants seen in cases of extensively drug-resistant *M. tuberculosis* [2,10]. These newly identified minority variants could represent novel molecular targets as helper drugs to increase the effectiveness of either the single or combinational therapies [70,71].

In conclusion, this was the first study to apply in-depth directed evolution methodology to *M. tuberculosis*, which allowed for the identification of population stepwise mutations supporting rifampicin and linezolid resistance. Here, variations in five genes were identified as being associated with rifampicin resistance, while three genes were identified for linezolid resistance. As expected, the major resistance mutations *rpoB* and *rplC*, conferring resistance to rifampicin and linezolid, respectively, were observed. However, an additional six genes were observed with novel mutations associated with resistance, with five presenting as subpopulations. Novel *ppsD*, *glpK* and *mptC* mutants were also identified in individual sequencing reads of antibiotic-resistant clinical isolates. Through utilization of *E. coli* as a model organism, *ppsD* and *ppsE* or orthologue mutations were phenotypically linked to low-level rifampicin resistance for the first time in both *M. tuberculosis* and *E. coli*. Thus, an antibiotic tolerance strategy common to multiple critically antibiotic-resistant bacterial species is identified as a clinically important target for further study.

## Supplementary material

10.1099/mgen.0.001723 Supplementary Material 1.

## Abbreviations

AF: allele frequency
CARD: comprehensive antibiotic resistance database
FACS: fluorescence-activated cell sorting
LZD: linezolid
MDR: multidrug-resistant
MIC: minimum inhibitory concentration
NCBI: National Center for Biotechnology Information
RIF: rifampicin
SNP: single nucleotide polymorphism
TB: tuberculosis
WGS: whole-genome sequencing
WHO: World Health Organization
XDR: extensively drug-resistant

## References

[1] WHO (2024). WHO Bacterial Priority Pathogen List, 2024.

[2] WHO (2024). Global Tuberculosis Report 2024.

[3] Naghavi M, Vollset SE, Ikuta KS, Swetschinski LR, Gray AP (2024). Global burden of bacterial antimicrobial resistance 1990–2021: a systematic analysis with forecasts to 2050. Lancet.

[4] Connolly LE, Edelstein PH, Ramakrishnan L (2007). Why is long-term therapy required to cure tuberculosis?. PLoS Med.

[5] Nguyen L (2016). Antibiotic resistance mechanisms in *M. tuberculosis*: an update. Arch Toxicol.

[6] Swain SS, Sharma D, Hussain T, Pati S (2020). Molecular mechanisms of underlying genetic factors and associated mutations for drug resistance in *Mycobacterium tuberculosis*. Emerg Microbes Infect.

[7] Liu Q, Via LE, Luo T, Liang L, Liu X (2015). Within patient microevolution of Mycobacterium tuberculosis correlates with heterogeneous responses to treatment. Sci Rep.

[8] Ismail N, Omar SV, Peters RPH (2019). *In vitro* study of stepwise acquisition of *rv0678* and *atpe* mutations conferring bedaquiline resistance. Antimicrob Agents Chemother.

[9] Ma Z, Lienhardt C, McIlleron H, Nunn AJ, Wang X (2010). Global tuberculosis drug development pipeline: the need and the reality. Lancet.

[10] Maltempe FG, Caleffi-Ferracioli KR, do Amaral RCR, de Oliveira Demitto F, Siqueira VLD (2017). Activity of rifampicin and linezolid combination in *Mycobacterium tuberculosis*. *Tuberculosis*.

[11] Zaw MT, Emran NA, Lin Z (2018). Mutations inside rifampicin-resistance determining region of *rpoB* gene associated with rifampicin-resistance in *Mycobacterium tuberculosis*. J Infect Public Health.

[12] WHO (2021). Catalogue of mutations in *Mycobacterium tuberculosis* complex and their association with drug resistance.

[13] Khan AS, Phelan JE, Khan MT, Ali S, Qasim M (2021). Characterization of rifampicin-resistant *Mycobacterium tuberculosis* in Khyber Pakhtunkhwa, Pakistan. Sci Rep.

[14] McNeil MB, Dennison DD, Shelton CD, Parish T (2017). *In vitro* isolation and characterization of oxazolidinone-resistant *Mycobacterium tuberculosis*. Antimicrob Agents Chemother.

[15] Walker TM, Miotto P, Köser CU, Fowler PW, Knaggs J (2022). The 2021 WHO catalogue of *Mycobacterium tuberculosis* complex mutations associated with drug resistance: a genotypic analysis. *Lancet Microbe*.

[16] Du J, Gao J, Yu Y, Li Q, Bai G (2021). Low rate of acquired linezolid resistance in multidrug-resistant tuberculosis treated with bedaquiline-linezolid combination. Front Microbiol.

[17] Barilar I, Battaglia S, Borroni E, Brandao AP, Brankin A (2024). Quantitative measurement of antibiotic resistance in *Mycobacterium tuberculosis* reveals genetic determinants of resistance and susceptibility in a target gene approach. Nat Commun.

[18] Waller NJE, Cheung CY, Cook GM, McNeil MB (2023). The evolution of antibiotic resistance is associated with collateral drug phenotypes in *Mycobacterium tuberculosis*. Nat Commun.

[19] Cain AK, Boinett CJ, Barquist L, Dordel J, Fookes M (2018). Morphological, genomic and transcriptomic responses of *Klebsiella pneumoniae* to the last-line antibiotic colistin. Sci Rep.

[20] Schön T, Werngren J, Machado D, Borroni E, Wijkander M (2020). Antimicrobial susceptibility testing of *Mycobacterium tuberculosis* complex isolates - the EUCAST broth microdilution reference method for MIC determination. Clin Microbiol Infect.

[21] Palomino JC, Martin A, Camacho M, Guerra H, Swings J (2002). Resazurin microtiter assay plate: simple and inexpensive method for detection of drug resistance in *Mycobacterium tuberculosis*. Antimicrob Agents Chemother.

[22] Luber P, Bartelt E, Genschow E, Wagner J, Hahn H (2003). Comparison of broth microdilution, E Test, and agar dilution methods for antibiotic susceptibility testing of *Campylobacter jejuni* and *Campylobacter coli*. J Clin Microbiol.

[23] Hill EB, Wayne LG, Gross WM (1972). Purification of mycobacterial deoxyribonucleic acid. J Bacteriol.

[24] Alcock BP, Huynh W, Chalil R, Smith KW, Raphenya AR (2023). CARD 2023: expanded curation, support for machine learning, and resistome prediction at the comprehensive antibiotic resistance database. Nucleic Acids Res.

[25] Bradley P, Gordon NC, Walker TM, Dunn L, Heys S (2015). Rapid antibiotic-resistance predictions from genome sequence data for *Staphylococcus aureus* and *Mycobacterium tuberculosis*. Nat Commun.

[26] Phelan JE, O’Sullivan DM, Machado D, Ramos J, Oppong YEA (2019). Integrating informatics tools and portable sequencing technology for rapid detection of resistance to anti-tuberculous drugs. Genome Med.

[27] Coll F, McNerney R, Preston MD, Guerra-Assunção JA, Warry A (2015). Rapid determination of anti-tuberculosis drug resistance from whole-genome sequences. Genome Med.

[28] Rosenthal A, Gabrielian A, Engle E, Hurt DE, Alexandru S (2017). The TB portals: an open-access, web-based platform for global drug-resistant-tuberculosis data sharing and analysis. J Clin Microbiol.

[29] Hall MB, Lima L, Coin LJM, Iqbal Z (2023). Drug resistance prediction for *Mycobacterium tuberculosis* with reference graphs. Microb Genom.

[30] Horan KA, Viberg L, Ballard SA, Globan M, Wirth W (2025). Bringing tuberculosis genomics to the clinic: development and validation of a comprehensive pipeline to predict antimicrobial susceptibility from genomic data, accredited to ISO standards. *Lancet Digit Health*.

[31] Andrews S (2010). FastQC: a quality control tool for high throughput sequence data. https://www.bioinformatics.babraham.ac.uk/projects/fastqc.

[32] Bolger AM, Lohse M, Usadel B (2014). Trimmomatic: a flexible trimmer for Illumina sequence data. Bioinformatics.

[33] Li H, Durbin R (2009). Fast and accurate short read alignment with Burrows-Wheeler transform. Bioinformatics.

[34] Danecek P, Bonfield JK, Liddle J, Marshall J, Ohan V (2021). Twelve years of SAMtools and BCFtools. Gigascience.

[35] Seemann T (2015). https://github.com/tseemann/snippy.

[36] Robinson JT, Thorvaldsdóttir H, Winckler W, Guttman M, Lander ES (2011). Integrative genomics viewer. Nat Biotechnol.

[37] Seemann T (2014). Prokka: rapid prokaryotic genome annotation. Bioinformatics.

[38] Karp PD, Billington R, Caspi R, Fulcher CA, Latendresse M (2019). The BioCyc collection of microbial genomes and metabolic pathways. *Brief Bioinform*.

[39] Paysan-Lafosse T, Blum M, Chuguransky S, Grego T, Pinto BL (2023). InterPro in 2022. Nucleic Acids Res.

[40] Edgar RC (2004). MUSCLE: multiple sequence alignment with high accuracy and high throughput. Nucleic Acids Res.

[41] Tamura K, Stecher G, Kumar S (2021). MEGA11: Molecular Evolutionary Genetics Analysis Version 11. Mol Biol Evol.

[42] Wiegand I, Hilpert K, Hancock REW (2008). Agar and broth dilution methods to determine the minimal inhibitory concentration (MIC) of antimicrobial substances. Nat Protoc.

[43] Bisson GP, Mehaffy C, Broeckling C, Prenni J, Rifat D (2012). Upregulation of the phthiocerol dimycocerosate biosynthetic pathway by rifampin-resistant, *rpoB* mutant *Mycobacterium tuberculosis*. J Bacteriol.

[44] Gopal P, Yee M, Sarathy J, Low JL, Sarathy JP (2016). Pyrazinamide resistance is caused by two distinct mechanisms: prevention of coenzyme A depletion and loss of virulence factor synthesis. ACS Infect Dis.

[45] Folkvardsen DB, Thomsen VØ, Rigouts L, Rasmussen EM, Bang D (2013). Rifampin heteroresistance in *Mycobacterium tuberculosis* cultures as detected by phenotypic and genotypic drug susceptibility test methods. J Clin Microbiol.

[46] Jamieson FB, Guthrie JL, Neemuchwala A, Lastovetska O, Melano RG (2014). Profiling of *rpoB* mutations and MICs for rifampin and rifabutin in *Mycobacterium tuberculosis*. J Clin Microbiol.

[47] Maharjan R, Ferenci T (2017). The fitness costs and benefits of antibiotic resistance in drug-free microenvironments encountered in the human body. Environ Microbiol Rep.

[48] Patel Y, Soni V, Rhee KY, Helmann JD (2023). Mutations in *rpoB* that confer rifampicin resistance can alter levels of peptidoglycan precursors and affect β-Lactam susceptibility. mBio.

[49] Kaur D, Obregón-Henao A, Pham H, Chatterjee D, Brennan PJ (2008). Lipoarabinomannan of *Mycobacterium*: mannose capping by a multifunctional terminal mannosyltransferase. Proc Natl Acad Sci U S A.

[50] Yimcharoen M, Saikaew S, Wattananandkul U, Phunpae P, Intorasoot S (2022). The regulation of manlam-related gene expression in *Mycobacterium tuberculosis* with different drug resistance profiles following isoniazid treatment. Infect Drug Resist.

[51] Sheen P, Requena D, Gushiken E, Gilman RH, Antiparra R (2017). A multiple genome analysis of *Mycobacterium tuberculosis* reveals specific novel genes and mutations associated with pyrazinamide resistance. BMC Genomics.

[52] Shur KV, Zaychikova MV, Mikheecheva NE, Klimina KM, Bekker OB (2016). Draft genome sequence of *Mycobacterium tuberculosis* strain B9741 of Beijing B0/W lineage from HIV positive patient from Siberia. Genom Data.

[53] Safi H, Gopal P, Lingaraju S, Ma S, Levine C (2019). Phase variation in *Mycobacterium tuberculosis glpK* produces transiently heritable drug tolerance. Proc Natl Acad Sci U S A.

[54] Cole ST, Brosch R, Parkhill J, Garnier T, Churcher C (1998). Deciphering the biology of *Mycobacterium tuberculosis* from the complete genome sequence. Nature.

[55] Timm J, Bateson A, Solanki P, Paleckyte A, Witney AA (2023). Baseline and acquired resistance to bedaquiline, linezolid and pretomanid, and impact on treatment outcomes in four tuberculosis clinical trials containing pretomanid. *PLoS Glob Public Health*.

[56] An Q, Lin R, Yang Q, Wang C, Wang D (2023). Evaluation of genetic mutations associated with phenotypic resistance to fluoroquinolones, bedaquiline, and linezolid in clinical *Mycobacterium tuberculosis*: a systematic review and meta-analysis. J Glob Antimicrob Resist.

[57] DeJesus MA, Gerrick ER, Xu W, Park SW, Long JE (2017). Comprehensive essentiality analysis of the *Mycobacterium tuberculosis* genome via saturating transposon mutagenesis. mBio.

[58] Gagneux S, Long CD, Small PM, Van T, Schoolnik GK (2006). The competitive cost of antibiotic resistance in *Mycobacterium tuberculosis*. Science.

[59] Yang M, Guja KE, Thomas ST, Garcia-Diaz M, Sampson NS (2014). A distinct MaoC-like enoyl-CoA hydratase architecture mediates cholesterol catabolism in *Mycobacterium tuberculosis*. ACS Chem Biol.

[60] Kanvatirth P, Jeeves RE, Bacon J, Besra GS, Alderwick LJ (2019). utilisation of the prestwick chemical library to identify drugs that inhibit the growth of *Mycobacteria*. PLoS One.

[61] Goude R, Parish T (2008). The genetics of cell wall biosynthesis in *Mycobacterium tuberculosis*. Future Microbiol.

[62] Palmer AC, Kishony R (2013). Understanding, predicting and manipulating the genotypic evolution of antibiotic resistance. Nat Rev Genet.

[63] Bongaerts N, Edoo Z, Abukar AA, Song X, Sosa-Carrillo S (2022). Low-cost anti-mycobacterial drug discovery using engineered *E. coli*. Nat Commun.

[64] Cronan JE (2024). Unsaturated fatty acid synthesis in bacteria: mechanisms and regulation of canonical and remarkably noncanonical pathways. Biochimie.

[65] Karp PD, Paley S, Caspi R, Kothari A, Krummenacker M (2025). The ecocyc database (2025). EcoSal Plus.

[66] Moore LR, Caspi R, Boyd D, Berkmen M, Mackie A (2024). Revisiting the y-ome of Escherichia coli. Nucleic Acids Res.

[67] Santos RMS, Samelo J, Oliveira AC, Cordeiro MM, Mora MJ (2025). Interaction of the antibiotic rifampicin with lipid membranes. Biomolecules.

[68] MacDermott-Opeskin HI, Gupta V, O’Mara ML (2022). Lipid-mediated antimicrobial resistance: a phantom menace or a new hope?. Biophys Rev.

[69] Zheng Z, Parsons JB, Tangallapally R, Zhang W, Rock CO (2014). Discovery of novel bacterial elongation condensing enzyme inhibitors by virtual screening. Bioorg Med Chem Lett.

[70] Sullivan GJ, Delgado NN, Maharjan R, Cain AK (2020). How antibiotics work together: molecular mechanisms behind combination therapy. Curr Opin Microbiol.

[71] Woods RJ, Read AF (2023). Combination antimicrobial therapy to manage resistance. Evol Med Public Health.

