# Supplementary material for: Uncovering resistance pathways to first- and last-line antibiotics in Mycobacterium tuberculosis populations
Source: Microb Genom. 2026 May 19;12(5):001723. doi: 10.1099/mgen.0.001723 (PMC13186403; doi:10.1099/mgen.0.001723)
Supplement: Supplementary Material 1. [file mgen-12-01723-s001.pdf]

# Supplementary Materials

## Supplementary Methods

### *Escherichia coli* ortholog, media and growth conditions

*Mycobacterium tuberculosis* genes associated with rifampicin and linezolid resistance were assessed for resistance phenotypes using single-gene knockout mutants of *E. coli*. To determine which *E. coli* mutants were possible, non-essential *E. coli* orthologs were identified by aligning *M. tuberculosis* proteins sequences from UniProt against *E. coli* K-12 substr. MG1665 protein sequences using BLASTP and cross-checking their essentiality in EcoCyc [1]. Protein similarity was also assessed in InterPro [2]. *E. coli* strains and targeted transposon insertion mutant used in this study are outlined in Supplementary Table 2 [3]. Bacterial strains were stored in 25% glycerol frozen stock (-80°C). From the frozen stock, strains were streaked onto Mueller Hinton 2 agar (MH; Becton Dickinson; Agar 1.5% w/v; Sigma-Aldrich) plates. Plated bacteria were grown overnight (18 ± 2 hrs) at 37°C. Overnight liquid cultures of bacteria were grown in 5 mL of MH liquid media (50 mL falcon tube), inoculated with five representative colonies from the cultured MH plates, and incubated overnight at 37°C with shaking at 200 rpm. The resultant cultures produced stationary phase bacterial cells, that were used in subsequent experiments. Bacterial growth was quantified by measuring the optical density of 1 mL of 1:10 diluted liquid cultures at 600 nm ($OD_{600nm}$) with a BioPhotometer (Eppendorf) [4].

### Minimum inhibitory concentration and growth dynamics for *E. coli* mutants

The minimum inhibitory concentration (MIC) of rifampicin for *E. coli* mutants was determined using the broth microdilution method with each mutant tested in triplicate at each antibiotic concentration [4]. MIC of mutants was assessed across 2.5 to 60 µg/mL of rifampicin. Assays were prepared in a flat-bottomed 96-well plate (Corning), with antibiotic concentrations prepared using MH broth as the diluent. Overnight cultures were serially diluted to an $OD_{600nm}$ of 0.00125 and dispensed into wells for a final inoculum of ~5 x $10^5$ CFU/mL. $OD_{600nm}$ was measured at 18 hrs using a SPECTROstar Omega (BMG Labtech). MIC was determined as the lowest concentration that inhibited 100% of growth compared to the positive growth control [4].

Growth was also assessed in rifampicin supplemented media (MH) over a narrower range of 2 to 20 µg/mL. Growth plates, of two biological replicates and three technical replicates, were statically incubated at 37°C for 18-24 hrs in an Omnilog reader (Biolog) with readings taken every 15 minutes

at 590nm and reported in arbitrary Omnilog units (AOU). Experimental data was analysed in R v4.3.1. Outlying data, such as spontaneous mutants or single replicates with no growth, were removed and missing values were dealt with using imputation by mean. Growth curves and boxplots were constructed using the ggplot2 R package (v3.5.1) [5]. The area under the logistic curve (AUC) was calculated using growthcurver R package (v0.3.1) [6].

## Flow cytometric assessment of cell membrane permeability

Cell membrane permeability of *E. coli* mutants was assessed fluorometrically using SYTOX Green. The uptake of SYTOX Green, a membrane impermeant nucleic acid stain, correlates with the loss of membrane integrity [7]. Rifampicin (RIF) treated (8 µg/mL for 18 hrs) and untreated stationary phase cultures of *E. coli* mutants were prepared in triplicate and resuspended in phosphate buffered saline (PBS) to $OD_{600nm}$ = 0.1. Additionally, a heat-killed control culture was generated by incubating cells suspended in PBS at 72°C for 30 min (Supplementary Fig. 4) [8]. Samples were treated with 5 µM SYTOX Green (ThermoFisher Scientific, S7020) for 10 min at room temperature in the dark. These cells were then analysed by flow cytometry (BD FACSymphony™ A1 Cell Analyzer). Light scattering (forward; FSC and side; SSC) and fluorescence signals were generated by a 488 nm interrogating laser, with SYTOX Green excitation captured through a 530/30 nm bandpass emission filter (B530). To determine appropriate parameter settings and cell culture dilutions for single cell events, a pilot experiment was performed using SYTOX Green stained and unstained cultures. Detector voltages for FSC and SSC were adjusted to identify and gate a population of single cell events, followed by B530 detector voltages to ensure fluorescence signals from all samples were within the linear range of the B530 detector and to exclude cell-derived autofluorescence. A FSC event threshold was then set using blank PBS to exclude events derived from contaminants. These settings and gates were used for all subsequent experiments. Median fluorescence intensity from each replicate was used to compare cell membrane permeability of treated and untreated *E. coli* mutants.

# Supplementary Figures and Tables

**Supplementary Table 1.** *Mycobacterium tuberculosis* mutant strains developed through directed evolution experiments. DNA extractions quality and quantity, FastQC results detailing average read length and paired-end reads sequenced, and the mean coverage of the aligned reads for the parental and each resistant mutant strain of *M. tuberculosis*.

| | Sample ID* | Conc. (ng/µL) | 260/280 | Average read length (bp) | Number of paired-end reads | Mean coverage (depth) |
|---|---|---|---|---|---|---|
| **Parental** | H37Rv | 417.1 | 1.87 | 146 | 2.8M | 185x |
| **Rifampicin populations** | 0.31-RIF | 198 | 1.82 | 146 | 2.2M | 148x |
| | 0.63-RIF | 410 | 1.9 | 146 | 2.3M | 151x |
| | 2.5-RIF | 230 | 1.89 | 145.5 | 2.5M | 165x |
| | 5.0-RIF | 345 | 1.9 | 146 | 2.1M | 151x |
| | 10.0-RIF | 245 | 1.88 | 145.5 | 2.1M | 137x |
| **Linezolid populations** | 0.63-LZD | 249.3 | 1.95 | 146 | 1.9M | 124x |
| | 1.25-LZD | 254.9 | 1.92 | 145 | 2.4M | 159x |
| | 2.5-LZD | 329 | 1.82 | 145.5 | 2.7M | 176x |
| | 5.0-LZD | 299 | 1.9 | 146 | 2.5M | 163x |
| | 10.0-LZD | 245 | 1.9 | 146.5 | 0.6M | 42x |

* Resistant-mutants are referred to as X-RIF and Y-LZD, where X and Y denote the relative MIC for rifampicin and linezolid, respectively.

**Supplementary Table 2.** Keio collection [3] *Escherichia coli* mutants used in this study.

| Strains | Description | Resistance Tested |
|---|---|---|
| K-12 BW25113 | Wild-type, parental strain of Keio collection mutants | |
| K-12 BW25113 *ΔfabF* | Knockout mutant of *fabF*, an ortholog of *ppsD* and *ppsE* in *M. tuberculosis,* with functions in fatty acid elongation | Rifampicin |

**Supplementary Table 3.** Total number of single nucleotide polymorphisms (SNPs) detected in parental and resistant *M. tuberculosis* populations relative to the reference genome (H37Rv) and H37Rv parental strain consensus sequence, identified by the variant calling pipeline used in this study (see methods).

<table>
<tr><th rowspan="2"></th><th rowspan="2">Sample ID*</th><th colspan="2">Sampling conditions</th><th colspan="2">Total SNPs identified</th></tr>
<tr><th>Conc. (µg/mL)</th><th>Passage</th><th>Reference genome†</th><th>H37Rv parental‡</th></tr>
<tr><td>Parental</td><td>H37Rv</td><td>0</td><td>1</td><td>8</td><td>3</td></tr>
<tr><td rowspan="5">Rifampicin populations</td><td>0.31-RIF</td><td>0.31</td><td>2</td><td>7</td><td>6</td></tr>
<tr><td>0.63-RIF</td><td>0.63</td><td>3</td><td>9</td><td>5</td></tr>
<tr><td>2.5-RIF</td><td>2.5</td><td rowspan="3">5</td><td>7</td><td>4</td></tr>
<tr><td>5.0-RIF</td><td>5</td><td>7</td><td>7</td></tr>
<tr><td>10.0-RIF</td><td>10</td><td>9</td><td>4</td></tr>
<tr><td rowspan="5">Linezolid populations</td><td>0.63-LZD</td><td>0.63</td><td>1</td><td>8</td><td>5</td></tr>
<tr><td>1.25-LZD</td><td>1.25</td><td rowspan="2">3</td><td>11</td><td>4</td></tr>
<tr><td>2.5-LZD</td><td>2.5</td><td>9</td><td>5</td></tr>
<tr><td>5.0-LZD</td><td>5</td><td>4</td><td>9</td><td>7</td></tr>
<tr><td>10.0-LZD</td><td>10</td><td>6</td><td>10</td><td>5</td></tr>
</table>

* Resistant-mutants are referred to as X-RIF and Y-LZD, where X and Y denote the relative MIC for rifampicin and linezolid, respectively.
† H37Rv reference genome, GenBank: CP110619 (see methods)
‡ Consensus sequence of the aligned H37Rv parental strain to the H37Rv reference genome (see methods)

**Supplementary Table 4.** Comparison of all mutations detected in parental and resistant *M. tuberculosis* populations by various TB resistance variant detection pipelines. Allele frequencies of variants detected by only tbtAMR and TBProfiler are outlined in Supplementary Table 5.

| | **Mutation*†** | **Minimum inhibitory concentration‡** | **Allele frequency (%)** | **This study§** | **Clinical TB resistance variant detection pipelines‖** | | | | | |
|---|---|---|---|---|---|---|---|---|---|---|
| | | | | | **tbtAMR** | **TBProfiler** | **TBPortals** | **DrPRG** | **CARD TB Mutations** | **Mykrobe** |
| **Rifampicin** | *rpoB*$^{H445Y}$ | 4–64x | 98–100 | Y | Y | Y | Y | Y | Y | Y |
| | *Rv0052*$^{M63Dfs151X}$ | 0–2x | 5–21 | Y | N | N | N | N | N | N |
| | *ppsD*$^{H888Tfs941X}$ | 0–2x | 2–41 | Y | N | N | N | N | N | N |
| | *ppsE*$^{T430Yfs141X}$ | 0–2x | 12–17 | Y | N | N | N | N | N | N |
| | *ettA*$^{N40H}$ | 2x | 16 | N | Y | N | N | N | N | N |
| | *embB*$^{L172R}$ | 2x, 64x | 16–31 | N | Y | Y | N | N | N | N |
| | *embB*$^{S174R}$ | 64x | 12–24 | N | N | Y | N | N | N | N |
| | *mptC*$^{V321A}$ | 4–64x | 49–96 | Y | N | N | N | N | N | N |
| **Linezolid** | *rplC*$^{C154R}$ | 0.25–4x | 100 | Y | Y | Y | Y | Y | N | N |
| | *glpK*$^{V192Cfs61X}$ | 0.5–2x | 23–100 | Y | Y | Y | N | N | N | N |
| | *glpK*$^{W322R}$ | 2x | 12 | N | Y | Y | N | N | N | N |
| | *echA12*$^{G239R}$ | 4x | 94 | Y | N | N | N | N | N | N |

* The scope of mutations assessed in this study was limited to those resulting in amino acid changes.
† Mutations in superscript brackets represent changes in the amino acid sequence. 'X' indicates a stop codon and 'fs' indicates a frameshift mutation.
‡ Numbers in brackets represent the relative minimum inhibitory concentration (MIC) (µg/mL).
§ Pipelines used for this study: Variant calling pipeline (see methods) and Snippy
‖ Using default parameters
Y with green shading indicates identification of variant in corresponding pipeline.

**Supplementary Table 5.** Allele frequencies (%) of variants detected by tbtAMR [9] and TBProfiler [10, 11], using default parameters.

| | tbtAMR | | | | | | | TBProfiler | | | | | | |
|---|---|---|---|---|---|---|---|---|---|---|---|---|---|---|
| | Allele frequency (%) | | | | | | | Allele frequency (%) | | | | | | |
| **Rifampicin mutations†** | **Relative MIC*** (µg/mL) | **No Ab‡** (0) | **2x** (0.31) | **4x** (0.63) | **16x** (2.5) | **32x** (5.0) | **64x** (10.0) | **Relative MIC*** (µg/mL) | **No Ab‡** (0) | **2x** (0.31) | **4x** (0.63) | **16x** (2.5) | **32x** (5.0) | **64x** (10.0) |
| | *rpoB*$^{H445Y}$ | - | - | 98 | 100 | 100 | 99 | *rpoB*$^{H445Y}$ | - | - | 98 | 100 | 100 | 100 |
| | *ettA*$^{N40H}$ | - | 16 | - | - | - | - | *embB*$^{L172R}$ | - | 31 | - | - | - | 30 |
| | *embB*$^{L172R}$ | - | - | - | - | - | 12 | *embB*$^{S174R}$ | - | - | - | - | - | 24 |
| | **Passage** | 1 | 2 | 3 | 5 | | | **Passage** | 1 | 2 | 3 | 5 | | |
| | Allele frequency (%) | | | | | | | Allele frequency (%) | | | | | | |
| **Linezolid mutations†** | **Relative MIC*** (µg/mL) | **No Ab‡** (0.00) | **0.25x** (0.63) | **0.5x** (1.25) | **1x** (2.5) | **2x** (5.0) | **4x** (10.0) | **Relative MIC*** (µg/mL) | **No Ab‡** (0.00) | **0.25x** (0.63) | **0.5x** (1.25) | **1x** (2.5) | **2x** (5.0) | **4x** (10.0) |
| | *rplC*$^{C154R}$ | - | 100 | 100 | 100 | 100 | 100 | *rplC*$^{C154R}$ | 0 | 100 | 100 | 100 | 100 | 100 |
| | *glpK*$^{V192Cfs61X}$ | - | - | 100 | 100 | 100 | - | *glpK*$^{V192Cfs61X}$ | - | - | 23 | 50 | 74 | - |
| | *glpK*$^{W322R}$ | - | - | - | - | 12 | - | *glpK*$^{W322R}$ | - | - | - | - | 12 | - |
| | **Passage** | 1 | | 3 | | 4 | 6 | **Passage** | 1 | | 3 | | 4 | 6 |

* Minimum inhibitory concentration (MIC) repesents the relative MIC to those determined in liquid media. Numbers in brackets represent the concentration (µg/mL).

† Mutations in superscript represent changes in the amino acid sequence. ‘X’ indicates a stop codon and ‘fs’ indicates a frameshift mutation.

‡ No antibiotic (No Ab).

**Supplementary Table 6.** *Mycobacterium tuberculosis* clinical strains containing *glpK* mutation GGGGGGGT → GGGGGGGGT (573). Strains limited to published isolates only. Pre-XDR = resistant to rifampicin, isoniazid, and a fluoroquinolone.

| Accession | Strain | Resistance phenotype | Reference |
|---|---|---|---|
| OAK81941.1 | B9741 | Pre-XDR | [12] |
| CP023636.1 | TBDM2699 | Pyrazidimase sensitive, otherwise unknown | [13] |
| CP023575.1 | CSV5769 | Pyrazidimase resistant, otherwise unknown | [13] |
| CP130812.1 | MTb-Oman-321856 | Unknown | [14] |
| HE608151.1 | UT205 | Unknown | [15] |
| FR878060.1 | *africanum* GM041182 | Sensitive | [16] |

**Supplementary Table 7.** Non-essential *Escherichia coli* orthologs of genes of interest and their identity to corresponding *Mycobacterium tuberculosis* genes implicated in rifampicin (RIF) resistance. Similarity comparison using BLASTP and InterPro.

| | ***M. tuberculosis*** | | ***E. coli* K-12*** | | **BLASTP** | | **InterPro** |
|---|---|---|---|---|---|---|---|
| | **Gene** | **Product/Function** | **Gene** | **Product/Function** | **Percent Identity** | **Query Coverage** | **Shared** |
| **RIF** | *ppsD* | Polyketide synthase, lipid metabolism | *fabF* | 3-oxoacyl-[acyl-carrier-protein] synthase II, fatty acid biosynthesis | 26.42% | 23% | Domain(s): KS3_2 Function(s): Fatty acid biosynthesis |
| | *ppsE* | Polyketide synthase, lipid metabolism | *fabF* | | 29.97% | 19% | |

**E. coli* gene functions assigned from EcoCyc (https://ecocyc.org/).

**Supplementary Table 8.** Relative growth compared to growth control (GC) for wild-type (WT) *Escherichia coli* and Δ*fabF* mutant (%) across a rifampicin concentration range of 2.5-60 µg/mL, with minimum inhibitory concentration observed at 15 µg/mL for both.

| **Conc. (µg/mL)** | **60** | **40** | **30** | **20** | **15** | **10** | **7.5** | **5** | **3.75** | **2.5** | **GC** |
|---|---|---|---|---|---|---|---|---|---|---|---|
| **WT** | 0% | 0% | 0% | 0% | 0% | 20% | 33% | 64% | 77% | 94% | 100% |
| **Δ*fabF*** | 0% | -1% | -1% | -1% | 0% | 34% | 48% | 55% | 56% | 69% | 100% |

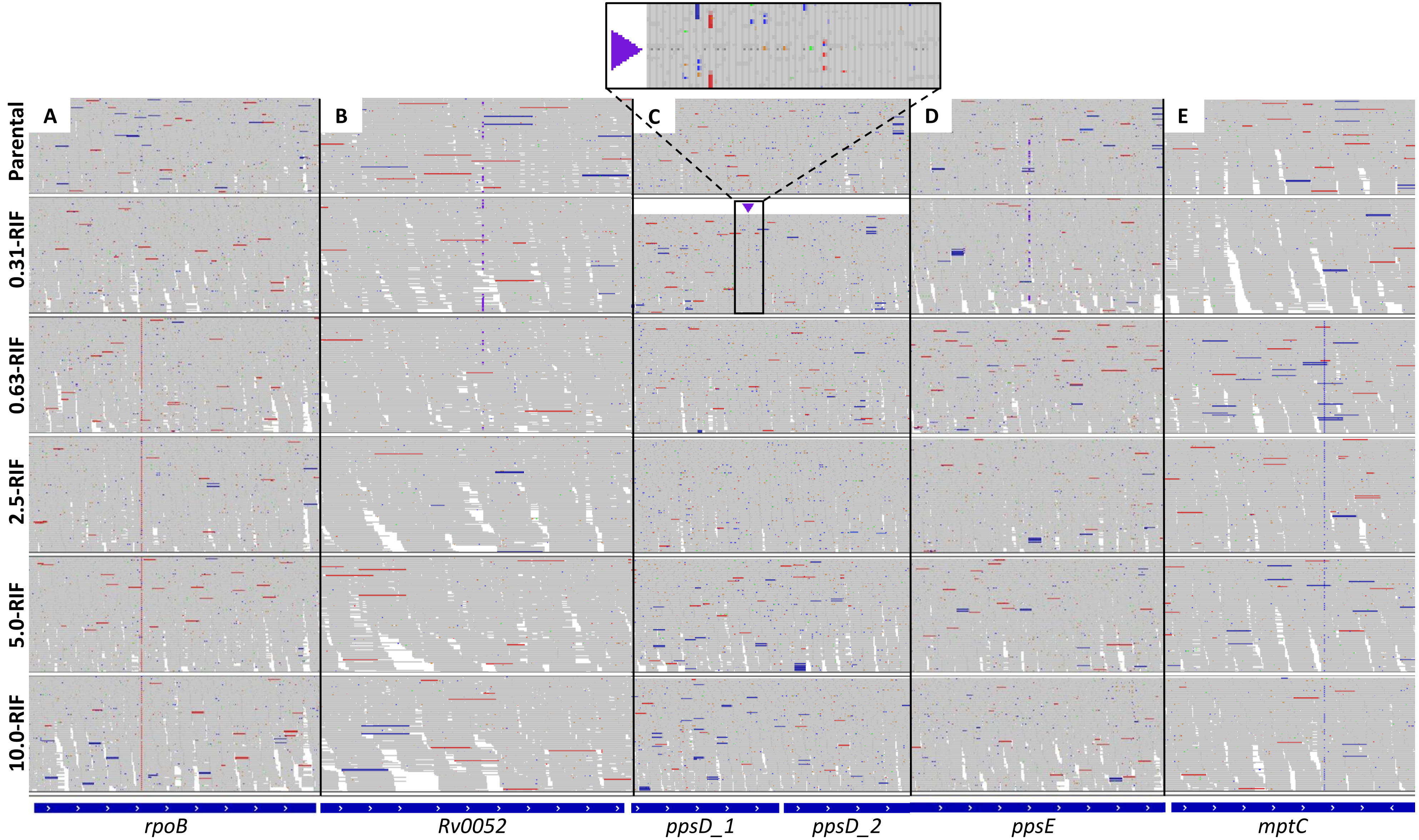


**Supplementary Figure 1.** Visualisation of rifampicin resistant mutant variants. A) *rpoB* single nucleotide polymorphism (SNP) B) *Rv0052* indel, C) *ppsD* indel, D) *ppsE* indel, and E) *mptC* (Rv2181) SNP. Zoomed callout and purple arrow are used to improve visualisation of *ppsD* indel.

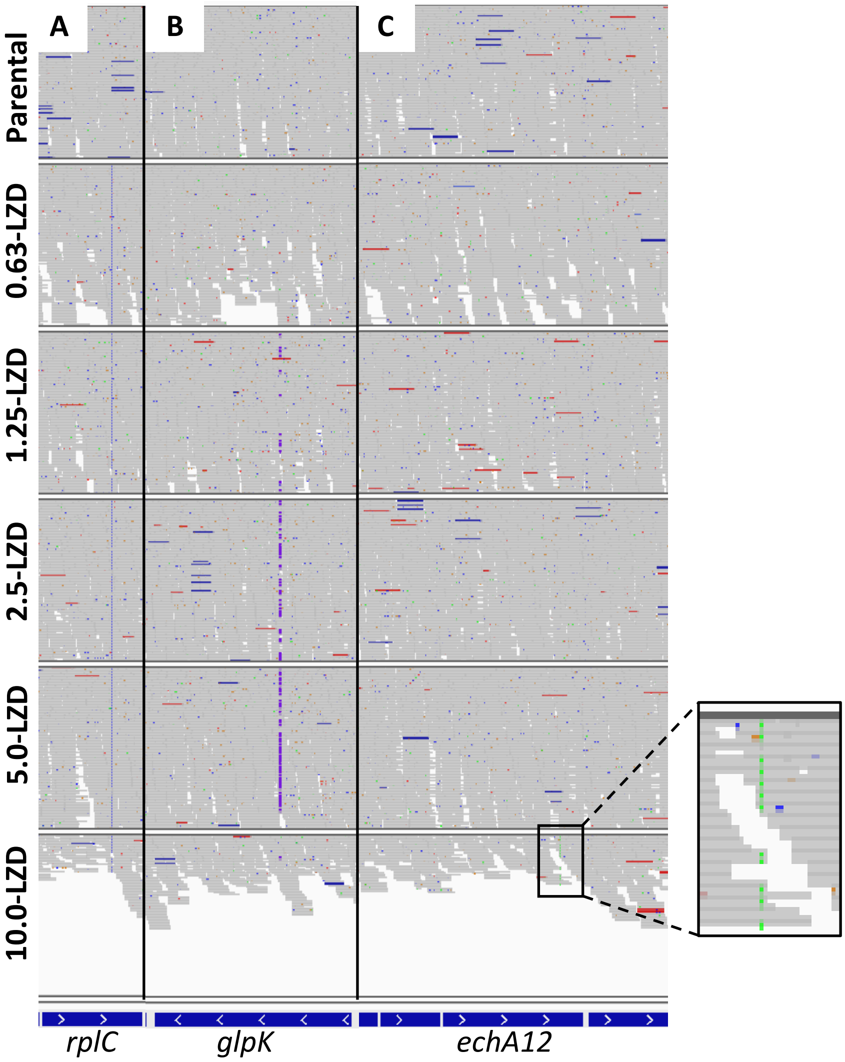


**Supplementary Figure 2.** Visualisation of linezolid resistant mutant variants. A) *rplC* single nucleotide polymorphism (SNP) B) *glpK* indel, and C) *echA12* SNP. Zoomed callout is used to improve visualisation of *echA12* SNP.

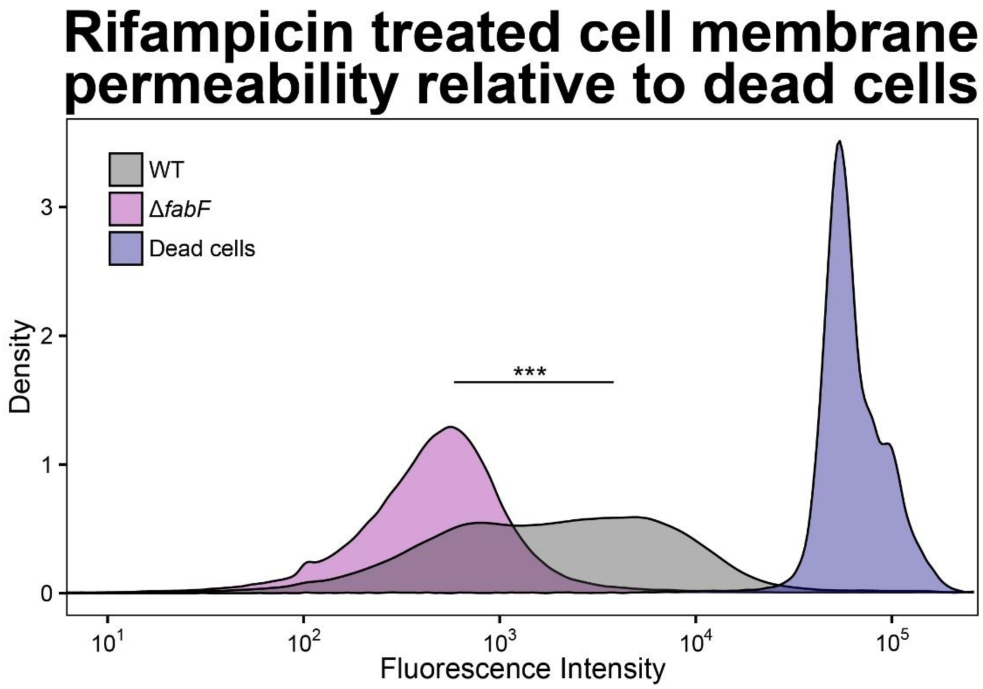


**Supplementary Figure 3.** Membrane permeability of wild-type *Escherichia coli* K12 and Δ*fabF* mutant relative to heat treated dead cells. Only approximately 7% of the wild-type population falling into the dead cell range based on dead cell gating. Each curve shows ~50,000 cells. Differences between wild-type and Δ*fabF* mutant were statistically analysed using a *t*-test, where median fluorescence intensity difference was used for fluorescence data. P-value: * $p < 0.05$, ** $p < 0.01$, *** $p < 0.001$, and **** $p < 0.0001$.

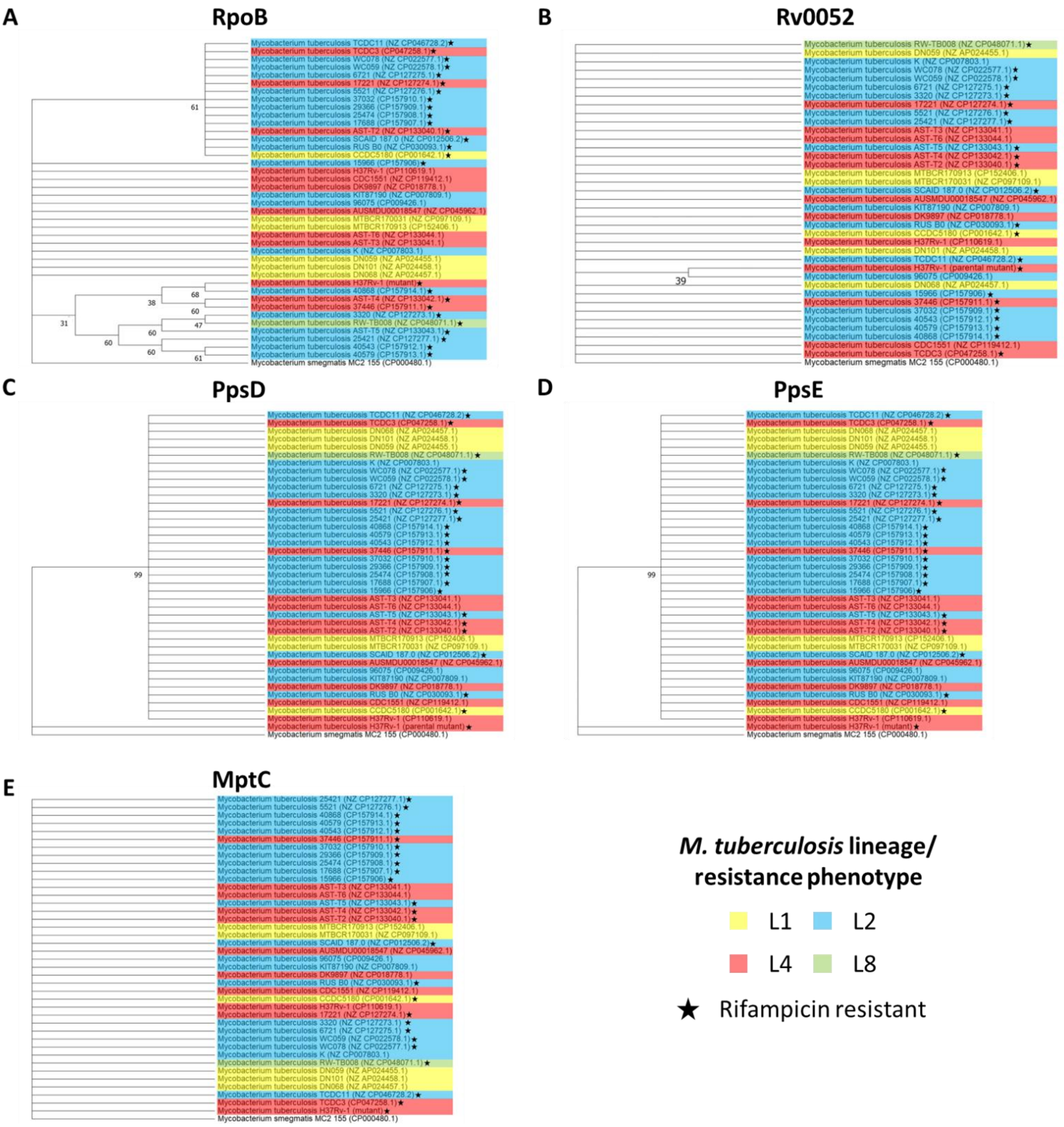


**Supplementary Figure 4.** Maximum likelihood tree of protein domains featuring the novel mutations aligned to previous clinical *Mycobacterium tuberculosis* strains. Protein domains for A) RpoB, B) Rv0052, C) PpsD, D) PpsE, and E) MptC aligned with 100 bootstrap replications using *Mycobacterium smegmatis* mc$^2$-155 rooted as outgroup. Branches collapsed if bootstrap support <30. Lineages highlighted as yellow for L1, blue for L2, red for L4, and green for L8. Rifampicin resistant isolates indicated by black star.

# Supplementary References

1. Keseler IM, Collado-Vides J, Gama-Castro S, Ingraham J, Paley S, Paulsen IT, et al. EcoCyc: a comprehensive database resource for *Escherichia coli*. *Nucleic Acids Res*. 2005;33(Database issue):D334-7. https://doi.org/10.1093/nar/gki108.
2. Paysan-Lafosse T, Blum M, Chuguransky S, Grego T, Pinto BL, Salazar GA, et al. InterPro in 2022. *Nucleic Acids Res*. 2023;51(D1):D418-d27. https://doi.org/10.1093/nar/gkac993.
3. Baba T, Ara T, Hasegawa M, Takai Y, Okumura Y, Baba M, et al. Construction of *Escherichia coli* K-12 in-frame, single-gene knockout mutants: the Keio collection. *Mol Syst Biol*. 2006;2:2006.0008. https://doi.org/10.1038/msb4100050.
4. Wiegand I, Hilpert K, Hancock RE. Agar and broth dilution methods to determine the minimal inhibitory concentration (MIC) of antimicrobial substances. *Nat Protoc*. 2008;3(2):163-75. https://doi.org/10.1038/nprot.2007.521.
5. Wickham H. ggplot2: Elegant Graphics for Data Analysis: Springer-Verlag New York; 2016.
6. Sprouffske K, Wagner A. Growthcurver: an R package for obtaining interpretable metrics from microbial growth curves. *BMC Bioinformatics*. 2016;17:172. https://doi.org/10.1186/s12859-016-1016-7.
7. Zand E, Schottroff F, Schoenher C, Zimmermann KS, Zunabovic-Pichler M, Jaeger H. Single-staining flow cytometry approach using SYTOX™ green to describe electroporation effects on *Escherichia coli*. *Food Control*. 2022;132:108488.
8. Kerstens M, Boulet G, Tritsmans C, Horemans T, Hellings M, Delputte P, et al. Flow cytometric enumeration of bacteria using TO-PRO®-3 iodide as a single-stain viability dye. *J Lab Autom*. 2014;19(6):555-61. https://doi.org/10.1177/2211068214546745.
9. Horan KA, Viberg L, Ballard SA, Globan M, Wirth W, Bond K, et al. Bringing tuberculosis genomics to the clinic: development and validation of a comprehensive pipeline to predict antimicrobial susceptibility from genomic data, accredited to ISO standards. *Lancet Digit Health*. 2025;7(12):100939. https://doi.org/10.1016/j.landig.2025.100939.
10. Coll F, McNerney R, Preston MD, Guerra-Assunção JA, Warry A, Hill-Cawthorne G, et al. Rapid determination of anti-tuberculosis drug resistance from whole-genome sequences. *Genome Med*. 2015;7(1):51. https://doi.org/10.1186/s13073-015-0164-0.
11. Phelan JE, O'Sullivan DM, Machado D, Ramos J, Oppong YEA, Campino S, et al. Integrating informatics tools and portable sequencing technology for rapid detection of resistance to anti-tuberculous drugs. *Genome Med*. 2019;11(1):41. https://doi.org/10.1186/s13073-019-0650-x.
12. Shur KV, Zaychikova MV, Mikheecheva NE, Klimina KM, Bekker OB, Zhdanova SN, et al. Draft genome sequence of *Mycobacterium tuberculosis* strain B9741 of Beijing B0/W lineage

from HIV positive patient from Siberia. *Genom Data*. 2016;10:61-2. https://doi.org/10.1016/j.gdata.2016.08.001.

13. Sheen P, Requena D, Gushiken E, Gilman RH, Antiparra R, Lucero B, et al. A multiple genome analysis of *Mycobacterium tuberculosis* reveals specific novel genes and mutations associated with pyrazinamide resistance. *BMC Genomics*. 2017;18(1):769. https://doi.org/10.1186/s12864-017-4146-z.

14. Al-Jardani A, Al Yaquobi F, Adikaram C, Al Wahaibi A, Al-Balushi L, Al-Zadjali S, et al. Genomic and geospatial epidemiology of *Mycobacterium tuberculosis* in Oman: first national insight using whole genome sequencing. *Int J Infect Dis*. 2023;130 Suppl 1:S4-s11. https://doi.org/10.1016/j.ijid.2023.04.001.

15. Isaza JP, Duque C, Gomez V, Robledo J, Barrera LF, Alzate JF. Whole genome shotgun sequencing of one Colombian clinical isolate of *Mycobacterium tuberculosis* reveals DosR regulon gene deletions. *FEMS Microbiol Lett*. 2012;330(2):113-20. https://doi.org/10.1111/j.1574-6968.2012.02540.x.

16. Bentley SD, Comas I, Bryant JM, Walker D, Smith NH, Harris SR, et al. The genome of *Mycobacterium africanum* West African 2 reveals a lineage-specific locus and genome erosion common to the *M. tuberculosis* complex. *PLoS Negl Trop Dis*. 2012;6(2):e1552. https://doi.org/10.1371/journal.pntd.0001552.
